# Effectiveness and safety of emergency department-based streaming interventions for low-acuity utilizers - systematic review and meta-analysis

**DOI:** 10.1186/s12873-026-01488-w

**Published:** 2026-02-19

**Authors:** Felix Holzinger, Konrad Schmidt, David Legg, Daniela Krüger, Cornelia Wäscher, Sylwia Steinke, Martin Möckel, Christoph Heintze, Anna Slagman, Hendrik Napierala

**Affiliations:** 1 Charité – Universitätsmedizin Berlin, corporate member of Freie Universität Berlin and Humboldt-Universität zu Berlin, Institute of General Practice, Charitéplatz 1, 10117 Berlin, Germany; 2 Charité – Universitätsmedizin Berlin, corporate member of Freie Universität Berlin and Humboldt-Universität zu Berlin, Departments of Emergency Medicine, Campus Mitte and Virchow, Augustenburger Platz 1, 13353 Berlin, Germany; 3 Institute of General Practice, Faculty of Health Sciences Brandenburg, Brandenburg Medical School Theodor Fontane, Steinstraße 66/67, 14776 Brandenburg an der Havel, Germany

**Keywords:** Emergency department, Low-acuity patients, Patient streaming, Systematic review, Meta-analysis

## Abstract

**Background:**

Emergency department (ED) crowding is a global challenge, presumably aggravated by low-acuity utilization. Various patient streaming interventions have been implemented in EDs to reduce potentially unnecessary utilization and improve care coordination and patient throughput. This systematic review examined the effectiveness and safety of ED-based streaming for low-acuity patients.

**Methods:**

A search of MEDLINE, EMBASE, CINAHL, and Cochrane Library databases was conducted up to December 2, 2025. Screening and data extraction were performed in duplicate. RCTs, non-randomized controlled trials, interrupted time series, and before-after studies on general practitioner (GP) streaming, ED streaming, and urgent care (UC) streaming for low-acuity utilizers were eligible. We assessed outcomes related to care effectiveness, patient safety, and cost-effectiveness. Random-effects meta-analyses were performed. Risk of bias was assessed by the Effective Public Health Practice Project tool.

**Results:**

We included 137 publications reporting on 119 research projects. Meta-analyses showed higher proportions of cases managed in alternative tracks for GP streaming (0.32; CI 0.17;0.51) compared to ED streaming (0.25; CI 0.15;0.37). Both GP and ED streaming demonstrated reductions in length of stay, particularly for low-acuity patients (GP: SMD − 0.85; CI -1.37;-0.33; ED: SMD − 0.39; CI -0.56;-0.22). Safety outcomes, including leaving without being seen and unplanned ED reattendances, generally improved or were unchanged. The impact on ED utilization and cost-effectiveness remained inconclusive due to inconsistent evidence. There was high variability across outcomes, likely due to diverse context factors and multifaceted interventions.

**Conclusions:**

This review provides a comprehensive synthesis of various ED-based streaming interventions for low-acuity patients, with effect estimation by meta-analyses. Results suggest that GP and ED streaming improve care without compromising safety, with indications of a greater potential for alternative care with GP streaming. The predominance of observational studies with potential biases and unexplained heterogeneity, however, results in very low overall certainty of the evidence.

**Registration:**

PROSPERO CRD42022355935.

**Supplementary Information:**

The online version contains supplementary material available at 10.1186/s12873-026-01488-w.

## Background

Emergency department (ED) crowding is a persistent healthcare challenge [1, 2] and may be exacerbated by patients seeking care for low-acuity conditions [3]. Streaming interventions implemented in EDs aim to reduce potentially unnecessary utilization and improve patient flow [4, 5]. These include treatment by general practitioners (GP) in the ED [6, 7], streaming of patients to on-site urgent care (UC) clinics [8, 9], and ED process optimization, e.g., rapid assessment [10], or fast-tracking [11]. However, existing syntheses are inconclusive regarding effectiveness, safety, and cost-effectiveness [4, 6, 7, 9]. Likewise, the comparative effectiveness of different streaming approaches has not been comprehensively assessed more recently [4, 5], while there is much ongoing research and a growing evidence base.

This review aimed to assess the impact of ED-based streaming of low-acuity patients on relevant outcomes in the domains of care effectiveness, patient safety, and cost-effectiveness. It was part of the NODE project (Patient Navigation in German Emergency Care). The protocol was registered a priori in PROSPERO on 23rd September 2022 (ID CRD42022355935). Reporting adheres to PRISMA [12].

## Methods

### Methodological framework

This systematic review was guided by established evidence-based medicine frameworks, specifically adhering to core methodological principles and current standards endorsed by both the Cochrane Collaboration and JBI [13, 14].

### Populations and interventions

We focused on interventions aimed at ED populations with low-acuity complaints. This refers to individuals whose conditions, while they might be self-perceived as pressing, are classified as of lower urgency or as non-emergencies from a clinical perspective. We included research on care models designed to manage such cases in alternative pathways. This encompassed:

- GP streaming: steering to an alternative care pathway involving GPs, which could be organized as:
  - *Internal*: GP care in ED, usually after joint triage
  - *Co-located*: referral to on-site separate GP care venue
  - *External*: referral to GP treatment at another location
- ED streaming: within-ED process optimization and reorganization.
- UC streaming: steering to co-located UC facility.

Pre-hospital streaming without ED involvement was not studied. Studies that included patients of varying acuity levels were eligible if the intervention primarily targeted low-acuity patients. Where reported outcomes permitted, effects on the overall unselected ED population and on non-streamed patients (e.g., high acuity) could thus be determined additionally.

### Operationalization of “low-acuity”

Low-acuity is not a standardized concept and is operationalized and termed diversely in emergency care and research [15]. A common approach is triage-based categorization using established clinical scales like Emergency Severity Index (ESI), Manchester Triage System (MTS) or Canadian Triage and Acuity Scale (CTAS). Cut-offs typically define low-acuity as level 4 or 5 on these five-level scales. Other studies may use alternative criteria such as self-referral status or specific chief complaint categories. We accepted the definitions used within individual studies, reflecting the heterogeneous nature of international emergency care systems.

### Comparators and study designs

Comparators were standard ED care or other interventions. Eligible designs comprised randomized controlled trials (RCT), non-randomized controlled trials, interrupted time series, and before-after studies. All allow for comparison of either groups or time frames.

### Outcomes

Effectiveness outcomes comprised the potential for alternative care, ED utilization, process (waiting time, WT; length of stay, LOS) and care outcomes (hospital admissions, patient satisfaction, self-reported health status, subsequent ambulatory healthcare utilization). Safety outcomes considered were patients leaving without being seen (LWBS), unplanned ED reattendances and mortality. Cost-effectiveness was additionally evaluated.

“Potential for alternative care” was operationalized as the proportion of cases treated in the interventional compared to the standard pathway (applicable to the after period only in before-after studies). Studies in which group distributions were artificially influenced by design (e.g., matching) were not evaluated. For ED utilization, only studies considering underlying trends (e.g., interrupted time series) were useful to determine intervention impact.

### Literature search and screening

We searched MEDLINE, EMBASE, CINAHL, and the Cochrane Library. We used two search strategies centered at GP streaming interventions (strategy 1), and ED-based process optimization (strategy 2). No language or time restrictions were imposed. Searches were last updated on December 2, 2025 (during the peer review phase). See Appendix 1 for search syntax. Citation tracking, internet search, as well as preprint server (medRxiv, Research Square) and ClinicalTrials.gov screening were additionally performed. Two independent researchers reviewed titles and abstracts and then full texts. Disagreements were resolved by consensus or discussion with a third reviewer. For excluded studies and exclusion reasons (full text stage) see Appendix 2.

### Data extraction and risk of bias assessment

Two reviewers independently extracted study characteristics and outcome data on a REDCap [16] server. All results compatible with each outcome domain were collected. Disagreements were resolved through discussion with a third reviewer. Risk of bias (ROB) was assessed by the Effective Public Health Practice Project (EPHPP) Quality Assessment Tool [17] by two reviewers independently, discrepancies resolved by discussion with a third researcher.

### Data analysis and synthesis

Quantitative analyses were executed in R 4.0.1 (R Foundation for Statistical Computing, Vienna, Austria) with the ‘meta’ package [18]. For potential for alternative care, proportions were visualized, including a pooled estimate, by the ‘metprop’ function in a random effects model. A logit transformation was used. Between-study variance was estimated by the restricted maximum likelihood (REML) method. For other outcomes, effect sizes were calculated or extracted from publications. Standardized mean differences (SMD) for continuous outcomes were calculated from means and standard deviations, or by the Wan method [19] from medians and quantiles or ranges. Odds ratios (OR) for dichotomous outcomes were calculated from event numbers/rates. Random-effects meta-analyses using the inverse variance method with REML estimation of between-study variance were performed by the ‘metagen’ function [18]. Prediction intervals were calculated [20]. For heterogeneity investigation, subgroup analyses were performed if there were at least two groups containing a minimum of five studies each to avoid spurious findings and retain power [21, 22]. We additionally conducted post-hoc sensitivity analyses to assess the potential influence of pediatric-only studies on the pooled estimates. Meta-analyses for all outcomes where such studies were included were repeated separately for adult and pediatric target populations. Studies with mixed but predominantly adult populations were grouped as “adult”. Publication bias was explored by funnel plots of effect size and standard error for outcomes with a minimum of ten studies in respective pooled analyses [23]. Plots were inspected visually and Egger tests were performed. Certainty of evidence for meta-analyzed outcomes was assessed according to GRADE [24, 25].

## Results

### Body of identified literature, characteristics of included studies

**Fig. 1:**
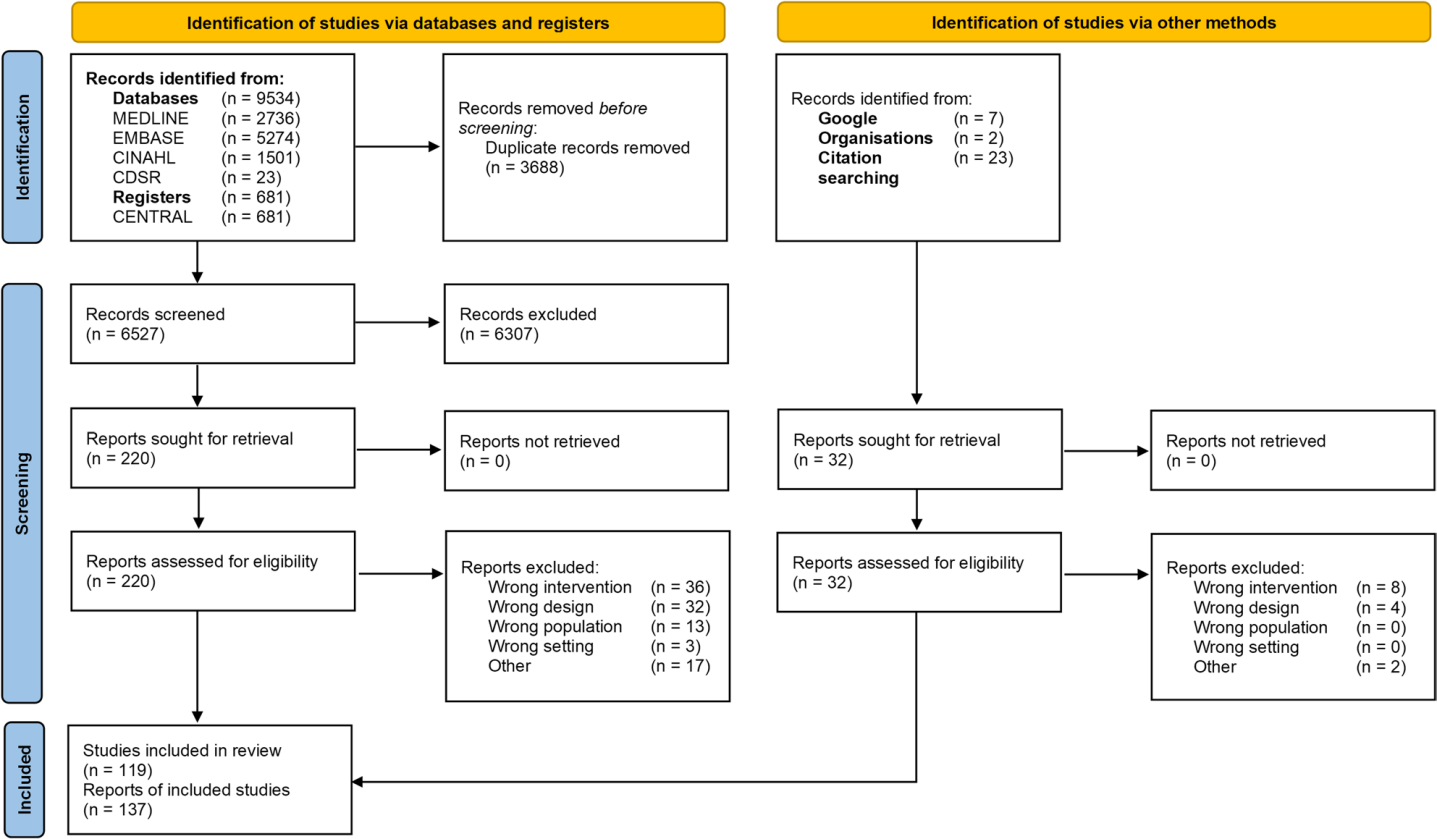
PRISMA flow chart. Source: Page MJ, et al. BMJ 2021;372:n71. doi: 10.1136/bmj.n71. Distributed under the terms of the Creative Commons Attribution License CC BY 4.0. To view a copy of this license, visit https://creativecommons.org/licenses/by/4.0/

After screening, 137 publications reporting on 119 research projects were included (Fig. 1, citations in Appendix 3). The greatest share originated from the U.S. (*n* = 41), followed by the Netherlands (*n* = 21) and the UK (*n* = 20). GP streaming was evaluated in 58 publications (42.3%); 71 (51.8%) were on ED streaming and two (1.5%) on UC streaming. Six reports (4.4%) concerned combinations of different approaches. Only one project was a parallel design RCT, one was a stepped-wedge cluster randomized trial, and nine were controlled clinical trials. This included studies with elements of randomization but predictable allocation. Of the remainder, three were cohort analytics, seven interrupted time series, and 98 had other designs (e.g., retrospective parallel, retrospective cohort). About a third of projects studied low-acuity patients only, whereas the majority reported on mixed urgency populations of which only part was targeted by streaming. Low-acuity was defined diversely by either triage categories (ESI *n* = 27, MTS *n* = 7, CTAS *n* = 12, Australasian Triage Scale *n* = 8; cut-offs frequently at 4 or 5 of the scales) or case criteria (e.g., self-referrals). For characteristics of included studies, see Table 1 in Appendix 4.

### Effectiveness: potential for alternative care

**Fig. 2:**
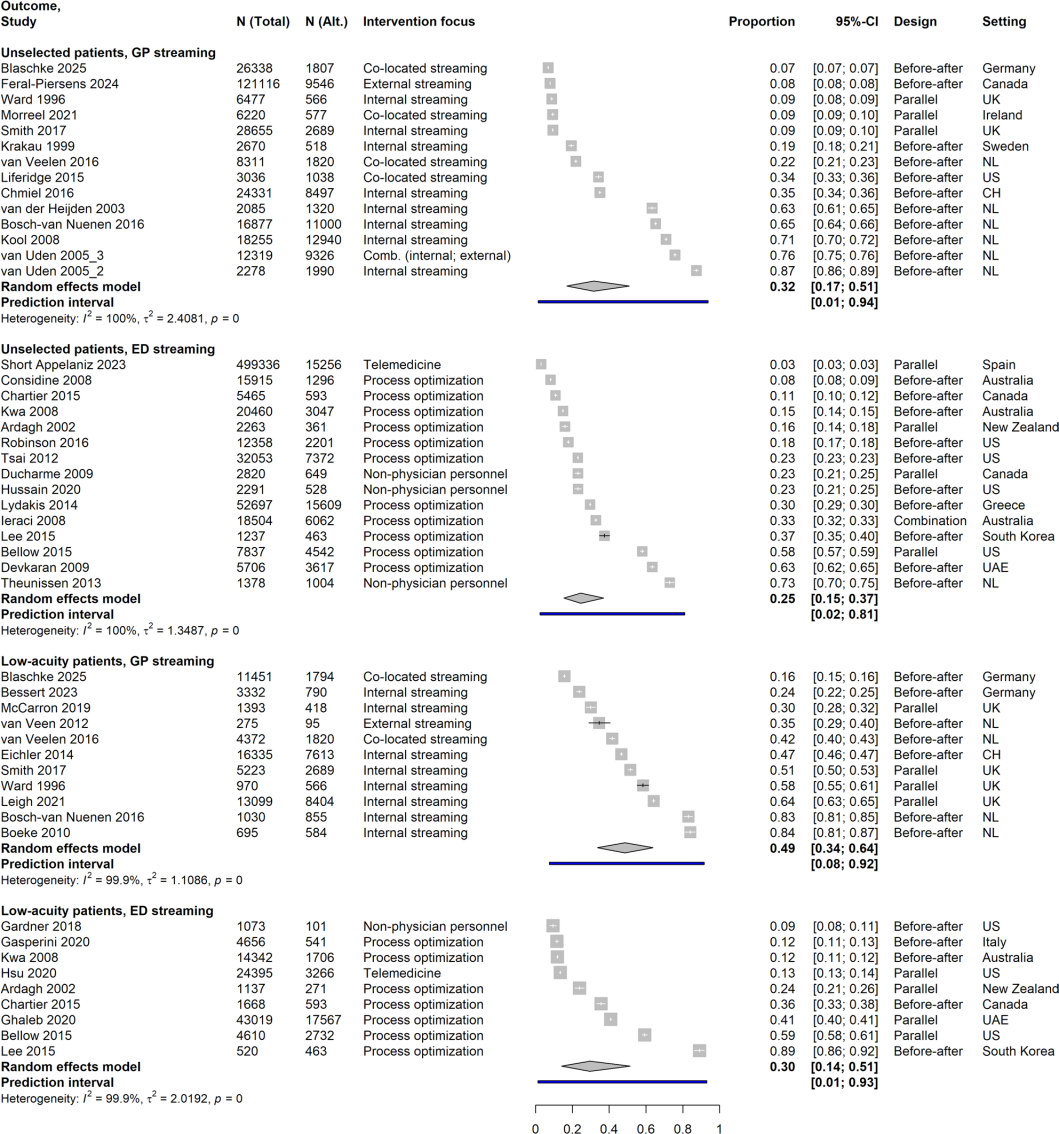
Pooled proportions of alternative management. Data is stratified according to reference population and streaming type; N (Alt.) = Number of patients treated in alternative care track; Intervention focus: for ED streaming, interventions with fast tracks, rapid assessment/triage, different assignment of personnel or modification of care environment were grouped together as “process optimization”; UK = United Kingdom; US = United States; NL = Netherlands; CH = Switzerland; UAE = United Arab Emirates

For unselected ED patients, GP streaming studies showed a higher pooled proportion of patients diverted, compared to ED streaming studies. Low-acuity patient populations generally had larger shares managed alternatively, GP streaming again showing higher rates. There was significant variability across studies, with wide ranges of reported proportions and broad prediction intervals (Fig. 2). UC streaming and combinations were not evaluated due to only one study reporting pertinent shares, respectively. Explorative subgroup analyses did not suggest explanations for heterogeneity. GP streaming in the Netherlands showed higher proportions of unselected ED patients diverted, compared to other countries. For low-acuity patients, reported reasons for treatment in standard care despite alternative options varied across studies, including medical criteria, refusal, and time restrictions in alternative stream availability. Variability across different healthcare systems, ED settings, and populations emphasizes the impact of local context factors and implementation specifics on alternative management capacities.

### Effectiveness: ED utilization

Information on potential effects of streaming on ED utilization varied across studies, and was frequently indirect, many reports mentioning ED census data without investigating intervention impact. Simple before-after studies prohibit causal attribution and are thus unhelpful. Interrupted time series and some parallel trials comparing settings provide relevant information, accounting for time trends and setting differences. Such yielded altogether mixed results, some showing decreased ED utilization (e.g [26, 27]) and others reporting increases (e.g [28, 29]) or no changes (e.g [30, 31]). Limited data comparability precluded meta-analysis.

### Effectiveness: process outcomes

#### Waiting time (WT)

**Fig. 3:**
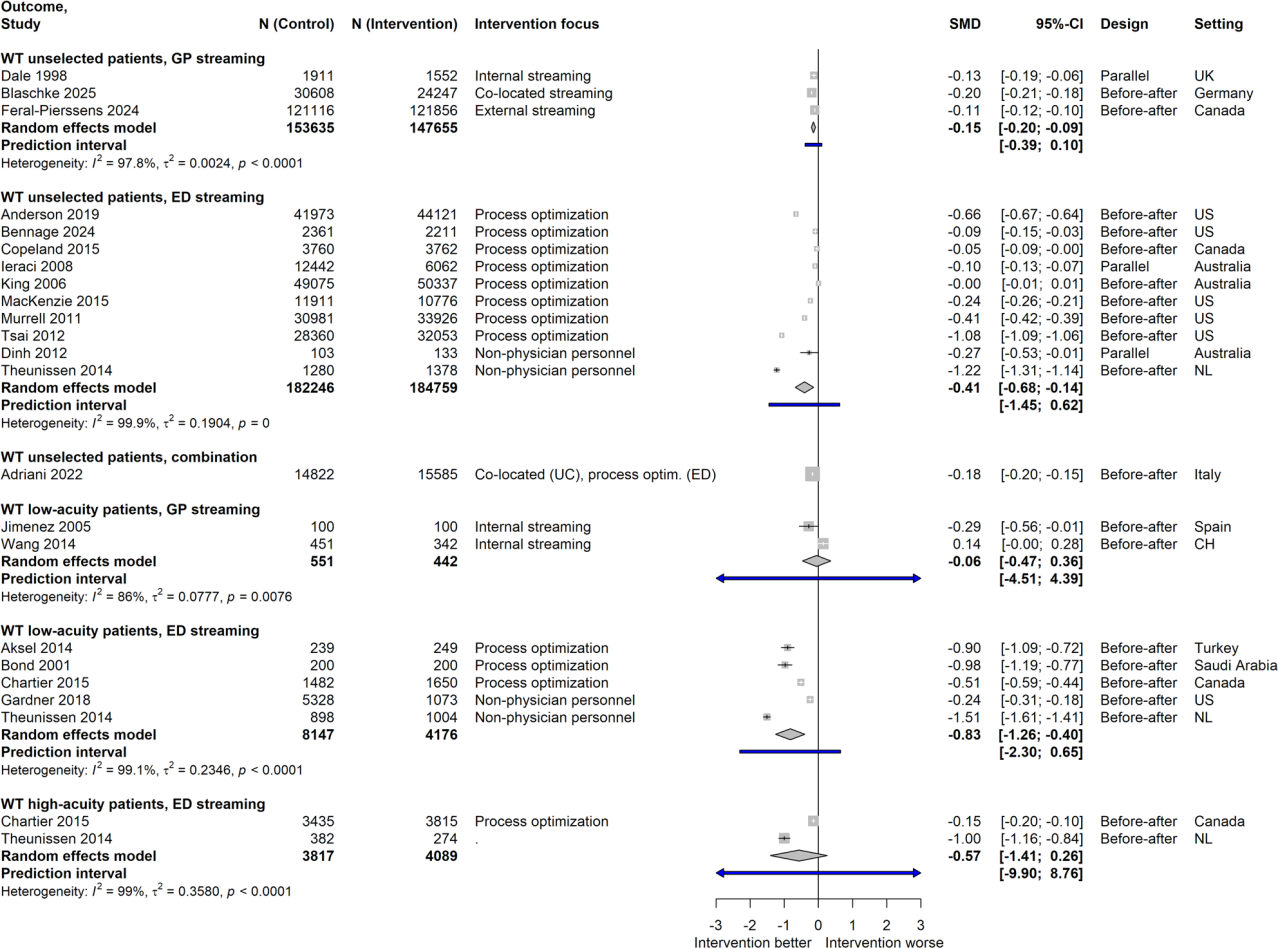
Effect sizes for waiting time. Data is stratified according to reference population and streaming type; WT = Waiting time; Intervention focus: for ED streaming, interventions with fast tracks, rapid assessment/triage, different assignment of personnel or modification of care environment were grouped together as “process optimization”; SMD = Standardized mean difference; UK = United Kingdom; US = United States; NL = Netherlands; CH = Switzerland

Meta-analyses showed mixed results across patient groups (Fig. 3). For WT of unselected ED patients of all urgency categories, three GP streaming studies allowed SMD calculation, with a significant but small pooled effect estimate. ED streaming studies indicated a moderate reduction in WT. High heterogeneity was observed in both analyses. For low-acuity patients, ED streaming studies – but not GP streaming interventions – showed a large decrease, again with high heterogeneity. Results for high-acuity patients were inconclusive. For all meta-analyses on WT, subgroup analyses were not reasonable due to small groups. Additional studies not included in meta-analyses mostly favored the interventional condition.

#### Length of stay (LOS)

**Fig. 4:**
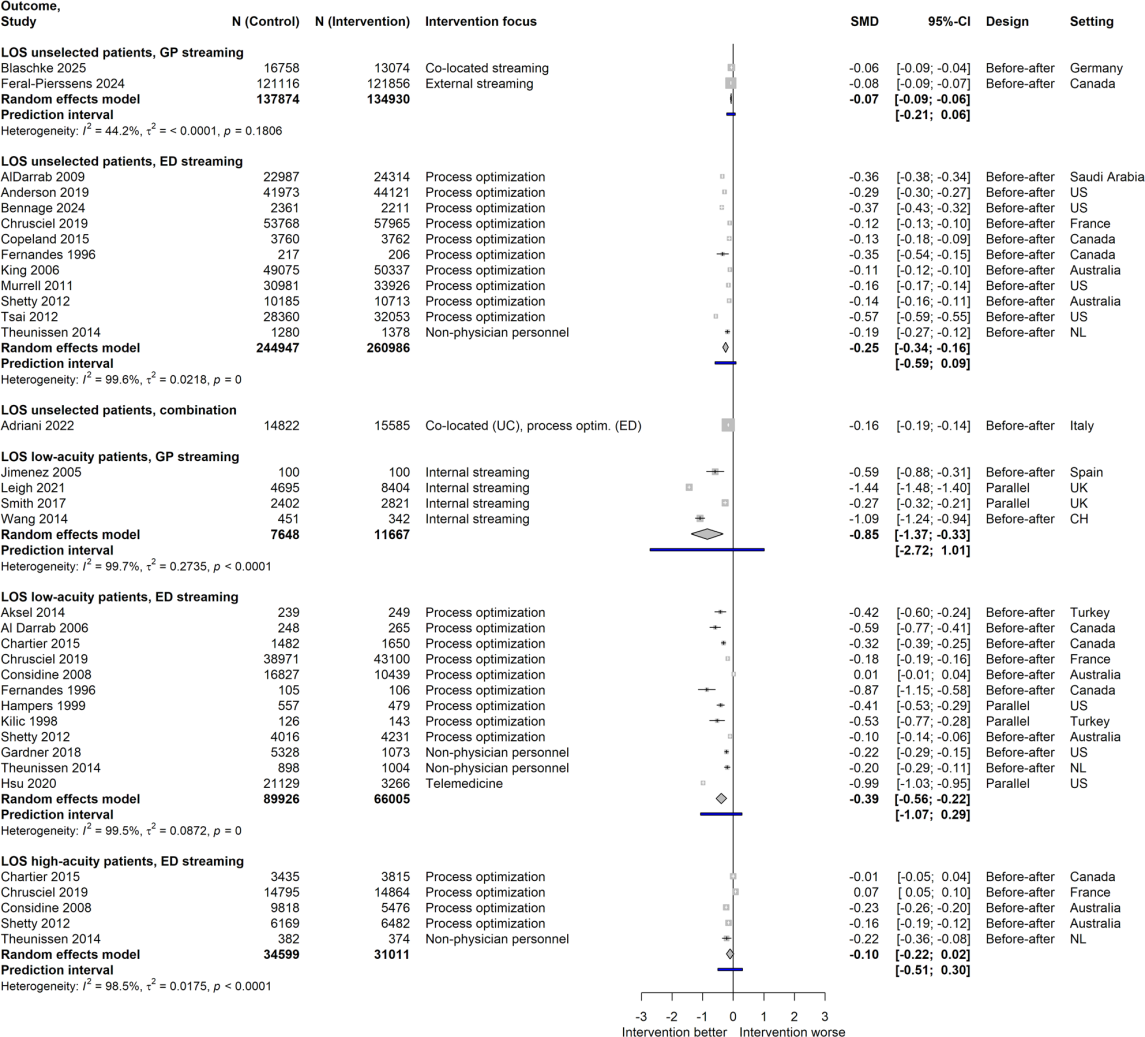
Effect sizes for length of stay. Data is stratified according to reference population and streaming type; LOS = Length of stay; Intervention focus: for ED streaming, interventions with fast tracks, rapid assessment/triage, different assignment of personnel or modification of care environment were grouped together as “process optimization”; SMD = Standardized mean difference; UK = United Kingdom; US = United States; NL = Netherlands; CH = Switzerland

GP streaming studies showed very small LOS reductions for unselected populations and large effects for low-acuity patients, while ED streaming demonstrated small but significant decreases for unselected ED patients and moderate reductions for low-acuity patients. No significant effects were found for high-acuity patients in ED streaming studies, with again no pertinent data for GP streaming available. Most analyses exhibited very high heterogeneity, suggesting that effectiveness is strongly influenced by context factors. Additional studies not included in meta-analyses generally supported these findings, favoring interventions or showing no difference. Due to small groups, heterogeneity could not be investigated by subgroup comparisons for GP streaming. Concerning ED streaming, such did not suggest an influence of study quality on LOS of low-acuity cases. Figure 4 presents the results.

### Effectiveness: care outcomes

#### Hospital admissions

**Fig. 5:**
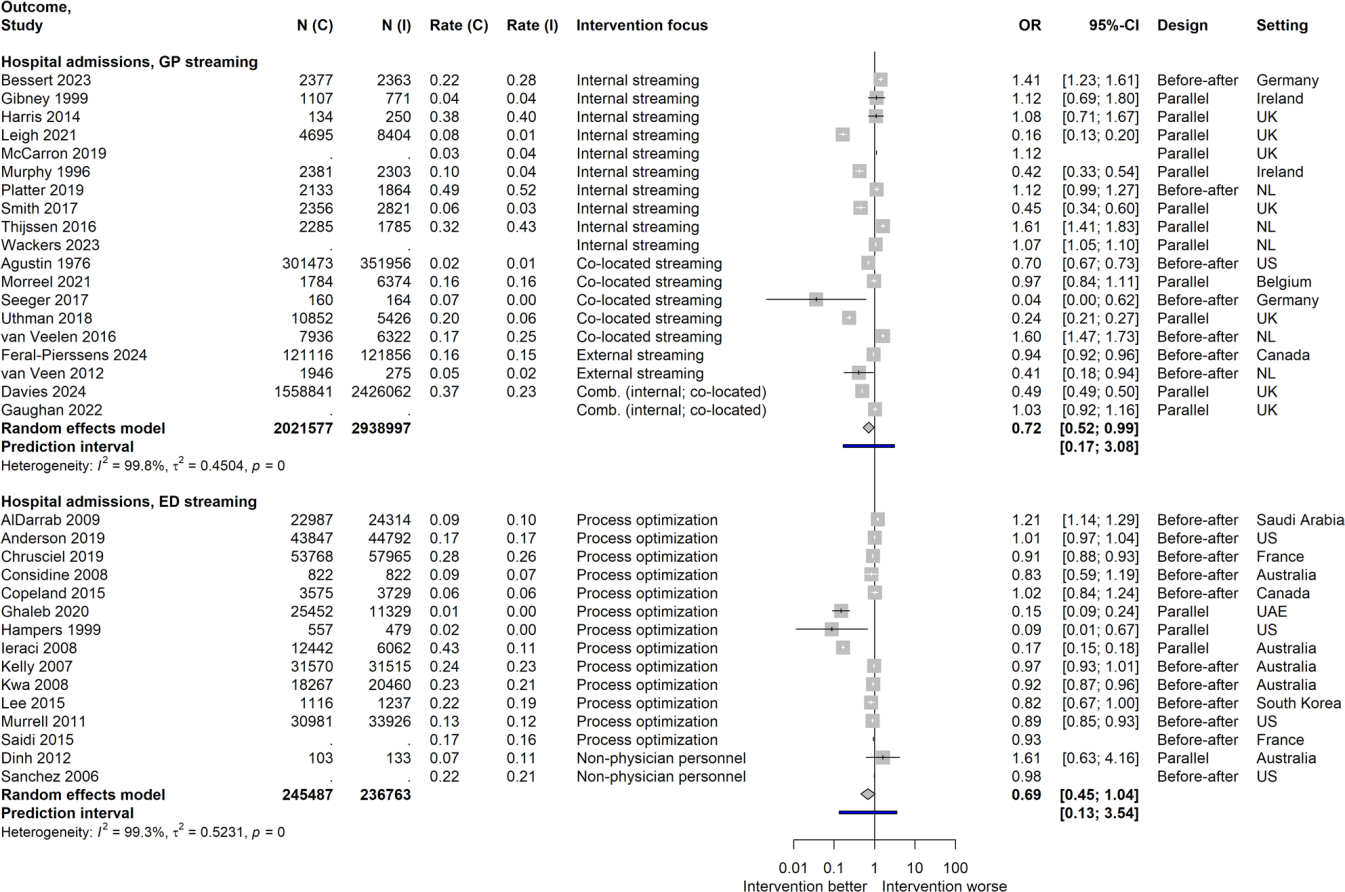
Effect sizes for hospital admissions. Data is stratified according to streaming type; N(I) = Population size in intervention; N(C) = Population size in control; Rate(I) = Hospital admission rate in intervention; Rate(C) = Hospital admission rate in control; Intervention focus: for ED streaming, interventions with fast tracks, rapid assessment/triage, different assignment of personnel or modification of care environment were grouped together as “process optimization; UK = United Kingdom; US = United States; NL = Netherlands; UAE = United Arab Emirates; Effect sizes of studies for which an OR but no confidence interval could be calculated are shown in the plot but not included in calculation of the pooled results

For GP streaming, a borderline significant pooled effect favored the intervention with fewer hospital admissions; individual study results varied. Subgroup analyses did not show significant differences for any marker investigated (internal vs. co-located streaming, parallel vs. before-after design, moderate vs. weak quality). ED streaming exhibited a similar effect magnitude, but it was non-significant. Subgroup analysis for study quality did not contribute to explaining between-study variability. Additional studies not included in pooling showed mixed results. For UC streaming, one study with data not permitting effect size calculation reported no significant differences. Effect sizes are illustrated in Fig. 5, including three studies not comprised in the meta-analyses, as only ORs – but no confidence intervals – were determinable.

#### Patient satisfaction, self-reported health status and subsequent ambulatory healthcare utilization

A total of 19 studies (six GP streaming, 13 ED streaming) provided information on patient satisfaction, using various measures, most frequently the Press Gainey instrument (Press Gainey Associates LLC, South Bend, Indiana, U.S.) or derivative scales (*n* = 6). However, use of Press Gainey scales was inconsistent (e.g., different items / scores chosen or reported). Of the remainder, none was used in more than one project. Results were diverse, with nine studies showing higher satisfaction in intervention groups, six reporting no differences, and four without statistical comparisons. No studies reported negative effects. Self-reported health status was assessed as rates of patients subjectively improved or cured. Limited evidence from four projects (one GP streaming, two ED streaming, one UC streaming) showed no significant differences between intervention and control. Evidence on subsequent ambulatory healthcare utilization from seven projects showed varied results, all but two reporting on GP streaming. The latter reported mixed findings, some showing no differences in short-term primary care consultations, while others indicated increased utilization in follow-up periods. UC streaming studies (*n* = 2) showed no significant effects or lower utilization compared to ED-treated patients. Altogether, available evidence very weakly suggests that GP streaming may be associated with increased ambulatory healthcare utilization in intermediate time frames. Meta-analysis was not feasible for any of these outcomes.

### Patient safety

#### Patients leaving without being seen (LWBS)

**Fig. 6:**
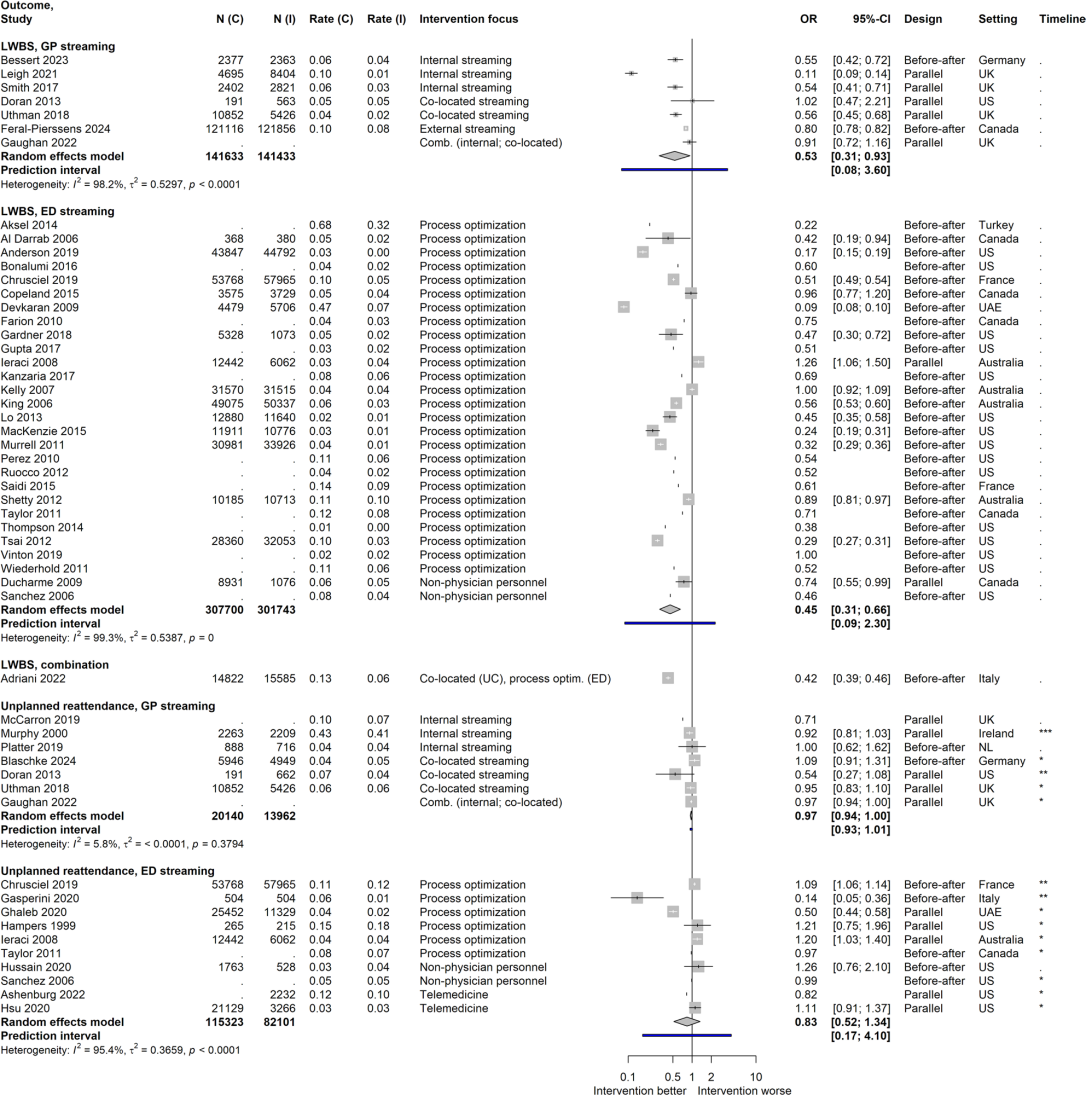
Effect sizes for patient safety outcomes (leaving without being seen and unplanned ED reattendances). Data is stratified according to streaming type; N(I) = Population size in intervention; N(C) = Population size in control; Rate(I) = Event rate in intervention; Rate(C) = Event rate in control; Intervention focus: for ED streaming, interventions with fast tracks, rapid assessment/triage, different assignment of personnel or modification of care environment were grouped together as “process optimization”; UK = United Kingdom; US = United States; NL = Netherlands; UAE = United Arab Emirates; Timeline (for unplanned ED reattendances only): + = Short term (up to seven days); +++ = Intermediate term (more than seven, up to 30 days); +++ = Long term (more than 30 days); Effect sizes of studies for which an OR but no confidence interval could be calculated are shown in the plot but not included in calculation of the pooled results

Meta-analyses showed significant LWBS rate reductions associated with both GP and ED streaming (Fig. 6). Data from a further 13 ED streaming studies permitted the determination of ORs but not confidence intervals (included in plot for illustration), all but one indicating lower odds of LWBS for the intervention, supporting pooled findings. A subgroup analysis for ED streaming showed no influence of study quality.

#### Unplanned ED reattendances

Meta-analysis of GP streaming studies (Fig. 6) showed non-significant results with a narrow prediction interval including the null effect. As to additional studies not included in the meta-analysis, one large GP streaming study reported a small but significant reduction in unplanned reattendances [31]. For ED streaming, pooled results were non-significant.

#### Mortality

None of twelve publications on GP and ED streaming reporting data showed any significant effects on mortality rates, irrespective of whether within-ED, short-term, or population-level mortality was considered. Due to very few events, heterogenous time frames, and no indication of effects in any individual study, meta-analysis was not performed.

### Cost-effectiveness

Six publications reported cost-effectiveness analyses, primarily focusing on GP streaming (*n* = 5). Evaluation methods (e.g., incremental cost-effectiveness ratios, cost-effectiveness planes) and results were diverse. Some studies found GP streaming cost-effective, with lower process times and costs compared to standard ED care, while others showed mixed or negative results. For example, one recent UK study with a very large dataset [28] found limited savings associated with outcomes changes, while another British project with a similar research aim [31] concluded that such were outweighed heavily by the cost of streaming services. The only analysis of UC streaming [32] likewise showed varied results depending on assumptions.

### Influence of pediatric studies and low-acuity definitions

Sensitivity analyses separating adult and pediatric populations provided important information regarding generalizability (for results, see Appendix 6, Figs. 11, 12, 13, 14, 15, 16, 17, 18, 19 and 20). Regarding potential for alternative care, consistent effects across populations could be demonstrated. Results on WT and LOS were likewise not impacted fundamentally, however the favorable effects on LOS seen for GP streaming of low-acuity patients did not attain statistical significance in a separate pooling of two pediatric-only studies. Concerning the impact of GP streaming on hospital admissions, the observed effect lost its significance when analyzing studies with adult populations separately, while it was significant in the pediatric sub-analysis. For GP streaming effects on patients leaving without being seen, exclusion of pediatric studies diminished the magnitude of the effect. An additional source of heterogeneity potentially stems from the lack of standardized low-acuity definitions across studies. We attempted subgroup comparisons of five-level triage scales versus alternative criteria, but no single approach was used frequently enough within individual outcomes to permit meaningful analyses.

### Risk of bias, publication bias, and certainty of the evidence

ROB assessment [17] covered six domains: selection bias, study design, confounders, blinding, data collection methods, and withdrawals and dropouts; for plots created with the robvis tool [33], see Figs. 7, 8, 9 and 10 in Appendix 5. Most studies showed low risk of selection bias, supporting population representativeness within their settings. However, the study design domain exhibited the highest risk, mainly due to retrospective before-after designs. Blinding was rarely formal but could occur inadvertently in study designs where new care tracks were implemented and compared to earlier care processes. Data collection generally had low ROB, utilizing clinical care process documentation. GP Streaming intervention studies showed a slightly better overall ROB profile. However, evidence quality in this research area can be judged as moderate to low.

Funnel plots and testing for asymmetry were exploratively performed for outcomes with a minimum of ten studies. Based on visual assessment and non-significant Egger tests, pooled effect estimates in our review appear not likely to be biased due to selective publication.

Certainty of evidence for meta-analyzed outcomes was assessed by GRADE for GP and ED streaming (Table 2 in Appendix 4). As per GRADE methods, “low certainty” is the highest initial judgment for evidence from non-randomized studies [34]. Evidence for all outcomes was downgraded to “very low certainty”, mainly due to high unexplained heterogeneity and potential ROB. For effects of GP streaming on LOS of unselected patients, downgrading was decided due to the pooled effect estimate stemming from only two individual studies. Very low certainty indicates insufficient evidence for firm conclusions and unknown true effects. Prediction intervals in the meta-analyses reflect this uncertainty.

## Discussion

### Summary of findings

GP streaming showed higher proportions managed in alternative tracks compared to ED streaming interventions, though the impact on ED utilization remains unclear. Care process metrics generally improved, with ED streaming reducing WT and LOS for both unselected and low-acuity ED patients, while GP streaming showed large reductions in LOS for low-acuity ED patients. Both streaming types reduced the likelihood of LWBS, and GP streaming showed borderline significant reductions of hospital admissions. Patient satisfaction either improved or showed no differences, and no significant effects on mortality were observed, while data was limited. Evidence on cost-effectiveness of GP streaming was inconclusive. The overall certainty of evidence for all outcomes was rated as very low due to high unexplained heterogeneity and potential risk of bias in the included studies.

## Results in context

### Effectiveness of GP streaming

There are very few quantifications of the potential for alternative management. In a systematic review on the impact of redirecting low-acuity patients including ED-based intervention studies, said potential had been estimated at a median of 85% [5]. However, only one GP streaming intervention was considered. Another publication [8] had reviewed GP cooperatives in the Netherlands, which were assessed as effective to reduce ED attendances. This aligns with our findings on high alternative care shares in the Netherlands, where the emergency care system has a strong primary care foundation and GP gatekeeping [35].

Regarding other outcomes, a UK report about initiatives to reduce WT and ED attendances found no evidence of effects on WT, based on five studies [4]. More recent evidence syntheses present a mixed picture. One [36] saw fewer admissions and lower resource use with GP streaming, while another [7] found such models frequently associated with an increase in attendances attributable to provider-induced demand, cost-effectiveness judged as doubtful as to high service costs.

A Cochrane review of GP streaming [6] included four trials and did not pool results as to high unexplained heterogeneity, authors assuming that this may have resulted from e.g., differences in design, triage criteria, or the healthcare system, aligning with our assumptions. The Cochrane group concluded that it is uncertain whether GP streaming reduces WT, LOS, or hospital admissions. A relatively recent narrative summary based on four literature reviews on effectiveness and safety of GP streaming [37], including [7] and [6], depicted evidence regarding flow improvements and economic effects still as limited. Authors emphasized the heterogeneous nature of services potentially contributing to inconsistent findings across studies.

Our sensitivity analyses suggest that some effects of GP streaming (as seen for hospital admissions and patients leaving without being seen) may be less pronounced in exclusively adult populations. However, this finding is based on few pediatric studies and there is no adequate data in the literature to support or refute it. Concerning conceivable explanations, divergent effects on hospital admissions may stem from different admission thresholds between adult and pediatric populations. However, this remains highly speculative.

### Effectiveness of ED streaming

Our findings generally support the conclusions of previous works. A systematic review of triage interventions [11] found that low-acuity fast-tracks reduced WT, LOS, and LWBS rates. Authors rated strength of evidence moderately strong, upgrading for outcome size and data concordance. Our discrepant assessment of evidence certainty highlights the interpretational variability regarding application of GRADE. Another research group [10] reviewed effectiveness of rapid assessment zones, finding consistent reductions in LOS and LWBS. Another evidence synthesis [38] of 21 limited quality studies focused on expanded nursing roles and alterations to triage processes likewise found a positive impact on LOS and LWBS. Building on a growing evidence base, a more recent systematic review [39] of 94 trials on interventions aimed at expediting ED throughput concluded that all intervention models reduced LOS, especially if focusing triage or incorporating fast tracking. While data was not pooled, results align well with our findings.

#### Effectiveness of UC streaming

There are few pertinent reviews which found no convincing evidence for walk-in UC centers reducing ED attendances [4, 8, 9] or improving patient outcomes [9].

#### Patient safety outcomes

We found both GP and ED streaming interventions associated with reductions in the odds of LWBS. For ED streaming, several reviews [10, 11, 38] – as mentioned – found evidence likewise suggestive of positive effects. While scarcely reviewed, none of the previous works suggested adverse effects on unplanned ED reattendances or mortality. For GP streaming, safety endpoints were not extensively covered previously.

#### Cost-effectiveness

Our inconclusive findings are consistent with the limited and mixed evidence reported in previous works (e.g [6, 7, 36]). This highlights an ongoing literature gap and the need for more robust economic evaluations. However, cost-effectiveness may be highly dependent on reimbursement regulations and cost structures in a particular healthcare setting.

#### What this review adds

This evidence synthesis extends previous findings, confirming potential benefits of GP and ED streaming on patient flow metrics while emphasizing the persistent challenges in synthesizing evidence in this complex area of healthcare services research. Our synthesis adds value through its comprehensive coverage of various streaming types and assessment of multiple dimensions of effectiveness and safety. The meta-analyses provide a unique contribution to scientific literature, as other reviews did not conduct quantitative syntheses. Despite important interpretational caveats, our approach allows for a more precise estimation of effects, concurrently highlighting the uncertainty of the evidence by providing prediction intervals throughout. We also provide a detailed analysis of alternative management potential which was not extensively covered or even quantified previously.

### Limitations

Many included studies were observational, often using retrospective before-after designs susceptible to bias. Combined with unexplained heterogeneity and potential ROB in the included studies, overall certainty of evidence is thus very low. While most studies showed low risk of selection bias within their settings, this does not translate to generalizability across healthcare systems with varying populations. Concurrently prediction intervals, while generally favoring the interventions, crossed the null effect threshold: while the overall trend is positive, the true effect in future implementations could potentially be null or even negative in some contexts. High I² values suggest that much of the observed variation is due to true differences between studies rather than chance. However, even small differences can inflate I² with large sample sizes. The very large populations of many included studies contributed to high precision of individual effect estimates with narrow confidence intervals.

Notwithstanding this, the high variability across outcomes likely stems from diverse local and healthcare system context factors. Additionally, interventions are often multifaceted, combining various care modifications. We must also again stress the heterogenous definition of low-acuity across included studies, with small group sizes limiting deeper investigations. This reflects the absence of international standardization for this concept, calling for greater consensus on operational definitions. While our inclusive approach was necessary to capture evidence across diverse healthcare settings, the divergent criteria represent a very important limitation and likely contribute substantially to the observed heterogeneity. Despite this “apples and oranges” situation common in health services research, statistical pooling remains valuable for illustration. It provides an overall estimate of effect direction and magnitude across diverse settings, offering a broader perspective than individual studies.

Several additional limitations warrant consideration. First, while we included all age groups as per our protocol, the small number of exclusively pediatric studies precluded formal subgroup analyses by age. Sensitivity analyses excluding pediatric studies showed minimal effects on pooled estimates, suggesting findings are largely applicable to adult populations, though generalizability to pediatric emergency care requires caution. Second, patient-reported outcomes, particularly satisfaction measures, could not be meta-analyzed due to diverse and inconsistently reported instruments. This underscores the value of core outcome set initiatives such as COMET [40] to improve comparability and synthesis of evidence in future studies.

## Conclusions

While GP and ED streaming show promise for improving care without increasing risks, effectiveness is likely context specific. Current evidence is insufficient for firm conclusions or definitive recommendations. The impact of streaming interventions on ED utilization, as well as their cost-effectiveness, remain unclear. Future studies should prioritize RCT or interrupted time series designs, use consistent core outcome measures, and provide comprehensive descriptions of interventions and context factors.

## Supplementary Information

Below is the link to the electronic supplementary material.

Supplementary Material 1: Appendix 1 - Search strategies.pdf. OVID MEDLINE search strategies

Supplementary Material 2: Appendix 2 - Excluded studies.pdf. List of excluded studies and exclusion reasons

Supplementary Material 3: Appendix 3 - Citations of included studies.pdf. Detailed list of included studies

Supplementary Material 4: Appendix 4 - Study characteristics and certainty of evidence tables.pdf. Tables detailing study characteristics and GRADE rating of evidence certainty. Captions for the tables in Appendix 4. Table 1: Characteristics of included studies. Table 2: Rating of evidence certainty (modified GRADE summary of findings table)

Supplementary Material 5: Appendix 5 - Risk of bias.pdf. Plots showing Effective Public Health Practice Project tool ratings

Supplementary Material 6: Appendix 6 - Sensitivity analyses.pdf. Forest plots of separate analyses for studies with adult and pediatric populations

## Data Availability

The data extracted and analyzed for the review are available from the corresponding author on reasonable request.

## Abbreviations

CI: Confidence Interval
CINAHL: Cumulative Index to Nursing and Allied Health Literature
CTAS: Canadian Triage and Acuity Scale
ED: Emergency Department
EMBASE: Excerpta Medica Database
EPHPP: Effective Public Health Practice Project
ESI: Emergency Severity Index
GP: General Practitioner
GRADE: Grading of Recommendations Assessment, Development and Evaluation
LOS: Length of Stay
LWBS: Leaving Without Being Seen
MEDLINE: Medical Literature Analysis and Retrieval System Online
MTS: Manchester Triage System
NODE: Patient Navigation in German Emergency Care
OR: Odds Ratio
PRISMA: Preferred Reporting Items for Systematic Reviews and Meta-Analyses
PROSPERO: International Prospective Register of Systematic Reviews
REML: Restricted Maximum Likelihood
RCT: Randomized Controlled Trial
ROB: Risk of Bias
SMD: Standardized Mean Difference
UC: Urgent Care
UK: United Kingdom
U.S.: United States of America
WT: Waiting Time
