# Supplementary material for: Effectiveness and safety of emergency department-based streaming interventions for low-acuity utilizers - systematic review and meta-analysis
Source: BMC Emerg Med. 2026 Feb 19;26:58. doi: 10.1186/s12873-026-01488-w (PMC12922365; doi:10.1186/s12873-026-01488-w)
Supplement: Supplementary file 1 — Supplementary Material 1: Appendix 1 - Search strategies.pdf. OVID MEDLINE search strategies [file 12873_2026_1488_MOESM1_ESM.pdf]

Appendix 1: Search strategies

Exemplary documentation of search strategies for OVID MEDLINE database, as performed on initial search run (update searches: identical syntax with time limit, restricting evidence retrieval to a period from the prior search).

OVID MEDLINE syntax for GP streaming interventions and cooperation models (strategy 1)

1. emergency Service, hospital/ or trauma centers/ or triage/

2. (emergency adj2 (care or healthcare or department? or unit? or room? or treatment? or patient? or cent$ or ward? or service? or facilit$ or clinic?)).ti,ab.

3. (accident adj2 emergency).ti,ab.

4. (triage adj2 (cent$ or department? or unit? or facilit$ or clinic?)).ti,ab.

5. (emergency adj2 (visit$ or consult$ or utili$ or attend$)).ti,ab.

6. (urgent adj2 (care or healthcare or "health care")).ti,ab.

7. or/1-6

8. (semiurgent or semi-urgent or nonemergen$ or non-emergen$ or non-urgent or nonurgent or "less urgent" or "low urgency" or "low-urgency" or "lower urgency" or "lower-urgency" or "low acuity" or "low-acuity" or "lower acuity" or "lower-acuity" or self-refer$ or (self adj refer$) or walk-in or (walk adj in) or avoidable or inappropriate$ or minor or ambulatory or "out of hours" or "out-of-hours" or "after hours" or "after-hours").ti,ab.

9. ((primary or general) adj7 (consultation? or problem? or need? or complaint? or symptom? or demand? or attend$ or treatable)).ti,ab.

10. or/8-9

11. after-hours care/ or ambulatory care facilities/ or community health centers/ or physicians' offices/ or general practitioners/ or physicians, family/ or physicians, primary care/ or outpatient clinics, hospital/

12. ((general or family or ambulatory or primary) adj3 (practitioner? or physician? or doctor? or professional? or practice)).ti,ab.

13. ((primary or ambulatory) adj2 (care or healthcare or "health care")).ti,ab.

14. (GP or GPs).ti,ab.

15. ((co-locat$ or adjacent or near$ or premises or ground? or site? or outside or integrat$) adj3 (hospital or emergency)).ti,ab.

16. ((collaborat$ or cooperat$ or co-operat$ or affiliat$ or associate$ or connect$ or linked) adj3 (community or non-hospital or nonhospital or practice? or cooperative? or co-operative? or center? or centre? or service? or unit? or facilit$ or clinic? or ambulatory)).ti,ab.

17. or/11-16

18. ((special$ or new or novel or innovat$ or alternative or reorgani$ or re-organi$) adj6 (care or healthcare or "health care" or treatment or setting? or track? or path? or pathway? or venue? or center? or centre? or service? or unit? or facilit$ or clinic? or model?)).ti,ab.

19. ((fast adj3 (care or treatment or track? or path? or pathway?)) or fast-track).ti,ab.

20. ((care or healthcare or "health care" or treatment) adj6 (track? or path? or pathway? or setting? or locus or model? or provision)).ti,ab.

21. (steer$ or navigat$ or diversion or divert$ or redirect$ or flow).ti,ab.

22. (demand or attend$).ti,ab.

23. or/18-22

24. randomized controlled trial/ or controlled clinical trial/ or pragmatic trial/ or multicenter study/ or non-randomized controlled trials as topic/ or interrupted time series analysis/ or controlled before-after studies/

25. (randomis$ or randomiz$ or randomly).ti,ab.

26. groups.ab.

27. (trial or multicenter or multi center or multicentre or multi centre).ti.

28. (intervention? or effect? or impact? or controlled or control group? or (before adj6 after) or (pre adj6 post) or ((pretest or pre test) and (posttest or post test)) or quasiexperiment$ or quasi experiment$ or evaluat$ or time series or time point? or repeated measur$ or versus).ti,ab.

29. or/24-28

30. exp animals/

31. humans/

32. 30 not (30 and 31)

33. (comment or news or editorial).pt.

34. comment on.cm.

35. or/32-34

36. 29 not 35

37. 36 and 7 and 10 and 17 and 23

OVID MEDLINE syntax ED-based process optimization interventions (strategy 2)

1. emergency Service, hospital/ or trauma centers/ or triage/

2. (emergency adj2 (care or healthcare or department? or unit? or room? or treatment? or patient? or cent$ or ward? or service? or facilit$ or clinic?)).ti,ab.

3. (accident adj2 emergency).ti,ab.

4. (triage adj2 (cent$ or department? or unit? or facilit$ or clinic?)).ti,ab.

5. (emergency adj2 (visit$ or consult$ or utili$ or attend$)).ti,ab.

6. or/1-5

7. (semiurgent or semi-urgent or nonemergen$ or non-emergen$ or non-urgent or nonurgent or "less urgent" or "low urgency" or "low-urgency" or "lower urgency" or "lower-urgency" or "low acuity" or "low-acuity" or "lower acuity" or "lower-acuity" or self-refer$ or (self adj refer$) or walk-in or (walk adj in) or avoidable or inappropriate$ or minor or ambulatory or "out of hours" or "out-of-hours" or "after hours" or "after-hours").ti,ab.

8. ((primary or general) adj7 (consultation? or problem? or need? or complaint? or symptom? or demand? or attend$ or treatable)).ti,ab.

9. ("low complexity" or "low-complexity" or "lower complexity" or "lower-complexity" or "uncomplicated" or "non-complex").ti,ab.

10. ((acuity or complexity) adj3 level?).ti,ab.

11. ((discharge$ or release$) adj3 likel$).ti,ab.

12. ("not" adj3 requir$).ti,ab.

13. or/7-12

14. ((fast adj3 (care or treatment or track? or path? or pathway? or assessment or evaluation or area or unit?)) or fast-track or fast-tracking).ti,ab.

15. (rapid adj3 (care or treatment or track? or path? or pathway? or assessment or evaluation or area or unit?)).ti,ab.

16. (expedit$ adj3 (care or treatment or track? or path? or pathway? or assessment or evaluation or area or unit?)).ti,ab.

17. (accelerate$ adj3 (care or treatment or track? or path? or pathway? or assessment or evaluation or area or unit?)).ti,ab.

18. ("see and treat" or "see-and-treat").ti,ab.

19. (patient? adj3 (stream$ or flow?)).ti,ab.

20. (split adj3 flow).ti,ab.

21. lean.ti,ab.

22. or/14-21

23. randomized controlled trial/ or controlled clinical trial/ or pragmatic trial/ or multicenter study/ or non-randomized controlled trials as topic/ or interrupted time series analysis/ or controlled before-after studies/

24. (randomis$ or randomiz$ or randomly).ti,ab.

25. groups.ab.

26. (trial or multicenter or multi center or multicentre or multi centre).ti.

27. (intervention? or effect? or impact? or controlled or control group? or (before adj6 after) or (pre adj6 post) or ((pretest or pre test) and (posttest or post test)) or quasiexperiment$ or quasi experiment$ or evaluat$ or time series or time point? or repeated measur$ or versus).ti,ab.

28. or/23-27

29. exp animals/

30. humans/

31. 29 not (29 and 30)

32. (comment or news or editorial).pt.

33. comment on.cm.

34. or/31-33

35. 28 not 34

36. 35 and 6 and 13 and 22
