# Supplementary material for: Effectiveness and safety of emergency department-based streaming interventions for low-acuity utilizers - systematic review and meta-analysis
Source: BMC Emerg Med. 2026 Feb 19;26:58. doi: 10.1186/s12873-026-01488-w (PMC12922365; doi:10.1186/s12873-026-01488-w)
Supplement: Supplementary file 2 — Supplementary Material 2: Appendix 2 - Excluded studies.pdf. List of excluded studies and exclusion reasons [file 12873_2026_1488_MOESM2_ESM.pdf]

Appendix 2: Excluded studies

Studies excluded in the full text screening stage, with exclusion reasons:

Wrong intervention: Numbers 1-44

1. Frank J, Shi K, Dunlop N, Cwinn A, Lee A. The effect of medical students on patient flow in a low acuity area of an academic emergency department. 2007 Society for Academic Emergency Medicine Annual Meeting. Acad Emerg Med. 14:S208-S.

2. Scott Bonham G, Barber GM. Use of Health Care Before and During Citicare. Med Care. 1987;25(2):111-9.

3. Green J, Dale J. Primary Care in Accident and Emergency and General Practice: A comparison. Soc Sci Med. 1992;35(8):987-95.

4. Fernandes CM, Price A, Christenson JM. Does reduced length of stay decrease the number of emergency department patients who leave without seeing a physician? J Emerg Med. 1997;15(3):397-9.

5. Docimo AB, Pronovost PJ, Davis RO, et al. Using the online and offline change model to improve efficiency for fast-track patients in an emergency department. Jt Comm J Qual Improv. 2000;26(9):503-14.

6. Chalder M, Sharp D, Moore L, Salisbury C. Impact of NHS walk-in centres on the workload of other local healthcare providers: time series analysis. BMJ (Clinical research ed). 2003;326(7388):532.

7. van Uden CJT, Crebolder HFJM. Does setting up out of hours primary care cooperatives outside a hospital reduce demand for emergency care? Emerg Med J. 2004;21(6):722-3.

8. Pickin DM, O'Cathain A, Fall M, Morgan AB, Howe A, Nicholl JP. The impact of a general practice co-operative on accident and emergency services, patient satisfaction and GP satisfaction. Fam Pract. 2004;21(2):180-2.

9. Chan TC, Killeen JP, Kelly D, Guss DA. Impact of rapid entry and accelerated care at triage on reducing emergency department patient wait times, lengths of stay, and rate of left without being seen. Ann Emerg Med. 2005;46(6):491-7.

10. O'Keeffe N. The effect of a new general practice out-of-hours co-operative on a county hospital accident and emergency department. Ir J Med Sci. 2008;177(4):367-70.

11. Mason S, O'Keeffe C, Knowles E, et al. A pragmatic quasi-experimental multi-site community intervention trial evaluating the impact of Emergency Care Practitioners in different UK health settings on patient pathways (NEECaP Trial). Emerg Med J. 2012;29(1):47-53.

12. Popovich MA, Boyd C, Dachenhaus T, Kusler D. Improving stable patient flow through the emergency department by utilizing evidence-based practice: one hospital's journey. J Emerg Nurs. 2012;38(5):474-8.

13. Gilligan P, Winder S, O'Kelly P. The reduce (reducing emergency department utilisation and crowding efforts) study. Acad Emerg Med. 2012;19(6):756.

14. Doyle SL, Kingsnorth J, Guzzetta CE, Jahnke SA, McKenna JC, Brown K. Outcomes of implementing rapid triage in the pediatric emergency department. J Emerg Nurs. 2012;38(1):30-5.

15. Murrell K, Offerman SR, Martinez J, Yee R. Use of an early patient-physician assignment system on emergency department arrival decreases time to physician and emergency department length of stay. Ann Emerg Med. 2012;60(4 SUPPL. 1):S50.

16. Enard KR, Ganelin DM. Reducing preventable emergency department utilization and costs by using community health workers as patient navigators. J Healthc Manag. 2013;58(6):412-28.

17. Leung A, Duic M, Gao D, Whatley S. Impact of process improvements on measures of emergency department efficiency. Can J Emerg Med. 2016;18(Suppl. 1):S29-S30.

18. Baliga S, Bitrus R, Krupp S, et al. Impact of evaluating patients in chairs on emergency department length of stay. Ann Emerg Med. 2016;68(4 Suppl. 1):S142.

19. O'Brien T, Wu R, Stanaitis I, Mukerji G, Rai M, Sabbah S. Acute ambulatory assessment to avoid admission (5alpha): A quality improvement study. J Gen Intern Med. 2017;32(2 Suppl. 1):S106.

20. Wing LK. Why AED Consulation Instead of Other Primary Halthcare Settings? A Study on 'Relationship between Utilization of Accident & Emergency Department and Other Primary Healthcare Settings'. Chinese Clinical Trial Register. 2018:ChiCTR1900021045.

21. Sanchez M, Suarez M, Asenjo M, Bragulat E. Improvement of emergency department patient flow using lean thinking. Int J Qual Health C. 2018;30(4):250-6.

22. Garrett JS, Berry C, Wong H, Qin H, Kline JA. The effect of vertical split-flow patient management on emergency department throughput and efficiency. Am J Emerg Med. 2018;36(9):1581-4.

23. Head RL, Monroe K, King A. Tuning up fast track: Refinements in triage criteria in a paediatric emergency room. J Investig Med. 2018;66(2):618.

24. Heinert SW, Mumford M, Kim SE, Hossain MM, Amashta ML, Massey MA. User Characteristics of a Low-Acuity Emergency Department Alternative for Low-Income Patients. West J Emerg Med. 2020;21(6):162-71.

25. Chacko J, Podlog M, Basile J, et al. Resource utilization of adult patients referred to the emergency department from an urgent care center. Hosp Pract (1995). 2020;48(5):272-5.

26. Barzin A, Seybold OC, Page C. Integrating an Urgent Care Clinic Into an Academic Family Medicine Practice. Fam Med. 2020;52(6):440-3.

27. University of Wisconsin. Wisconsin Evaluation of Emergency Department Care Coordination. ClinicalTrials.gov. 2020:NCT04550169.

28. Moore S, Young T, Irving A, Goodacre S, Brennan A, Amos Y. Controlled observational study and economic evaluation of the effect of city-centre night-time alcohol intoxication management services on the emergency care system compared with usual care. Emerg Med J. 2021;38(7):504-10.

29. Wang P, Bakshi S, Carlson L, et al. Fifteen minutes to change a health trajectory: Impact of a navigator intervention on primary care follow up and ed utilization in a medicaid patient population. J Gen Intern Med. 2021;36(Suppl. 1):S387-S8.

30. Hsieh A, Arena A, Oraha A, et al. Implementation of vertical split flow model for patient throughput at a community hospital emergency department. Acad Emerg Med. 2021;28(Suppl. 1):S25.

31. Schleef T, Engeleit K, Krause O, Schneider N. [Patients treated by general practitioners in a university emergency department before and after implementation of a triage system]. Med Klin Intensivmed Notfmed. 2022;(101575086).

32. Gilbert A, Brasseur E, Petit M, Donneau AF, D'Orio V, Ghuysen A. Advanced triage to redirect non-urgent Emergency Department visits to alternative care centers: the PERSEE algorithm. Acta Clin Belg. 2022;77(3):571-8.

33. Cotarelo AA, Hsieh A, Arena AW, et al. Implementation of Vertical Split Flow Model for Patient Throughput at a Community Hospital Emergency Department. West J Emerg Med. 2022;23(5.1):S1.

34. McLeod SL, Tarride JE, Mondoux S, et al. Health care utilization and outcomes of patients seen by virtual urgent care versus in-person emergency department care. Can Med Assoc J. 2023;195(43):E1463-E74.

35. Fernandes CM, Christenson JM. Use of continuous quality improvement to facilitate patient flow through the triage and fast-track areas of an emergency department. J Emerg Med. 1995;13(6):847-55.

36. Almeida A, Vales J. The impact of primary health care reform on hospital emergency department overcrowding: Evidence from the Portuguese reform. Int J Health Plann Manage. 2020;35(1):368-77.

37. Ebker-White A. The Sydney Triage to Admission Risk Tool (START) study: using a data analytics tool to drive early senior decision making and improve patient outcomes in Emergency Departments. Australian New Zealand Clinical Trials Registry. 2018: ACTRN12618000426280.

38. Arain M, Campbell MJ, Nicholl JP. Impact of a GP-led walk-in centre on NHS emergency departments. Emerg Med J. 2015;32(4):295-300.

39. Stoddart D, Ireland AJ, Crawford R, Kelly B. Impact on an accident and emergency department of Glasgow's new primary care emergency service. Health Bull. 1999;57(3):186-91.

40. Washington DL, Stevens CD, Shekelle PG, Brook RH. The effect of deferred care for nonemergent emergency department users: a randomized controlled trial. Abstr Book Assoc Health Serv Res Meet. 1999;16:187.

41. Pak A. HSD13 The Impact of Co-Locating GP-Led Minor Injury Clinic on Pediatric Emergency Department. Value Health. 2023;26(12 Suppl.):S296.

42. Hamer D, Jones GN, Loewe MR, Musso MW. Impact on an Urgent Care Clinic of a New Freestanding Emergency Department in a Resource-Scarce Area. Ochsner J. 2022;22(3):211-217.

43. ALJohani AA, Alhazmi JM, Alsaedi OH, Al-Ahmadi AF, Alshammary NS. Impact of urgent care centers on emergency department visits in Al Madina Al Munawara: A pre-post study. Saudi Med J. 2025;46(1):65-70.

44. Jimenez MLCD, Carascal MB, Figueras MD, et al. Impact of physician group practice in the operations, quality of care, and service satisfaction in the non-urgent section of an emergency department in a tertiary hospital in the Philippines: a mixed methods study. Int J Emerg Med. 2025;18(1):56.

Wrong study design: Numbers 45-80

45. Bentzen N, Christiansen T, Pedersen KM. A study of out-of-hours injury treatment. III. Possibilities for reorganization of treatment of injuries. Ugeskrift for Laeger. 1984;146(12):901-6.

46. Baker MG, Kljakovic M. The effect of emergency department policy change on Hutt district general practices. N Z Med J. 1992;105(942):380-3.

47. Roberts E, Mays N. Can primary care and community-based models of emergency care substitute for the hospital accident and emergency (A & E) department? Health Policy. 1998;44(3):191-214.

48. Murphy AW. 'Inappropriate' attenders at accident and emergency departments II: health service responses. Fam Pract. 1998;15(1):33-7.

49. McGugan EA, Morrison W. Primary Care or A&E? a Study of Patients Redirected from an Accident & Emergency Department. Scott Med J. 2000;45(5):144-7.

50. Combs S, Chapman R, Bushby A. Fast Track: one hospital's journey. Accid Emerg Nurs. 2006;14(4):197-203.

51. Combs S, Chapman R, Bushby A. Evaluation of Fast Track. Accid Emerg Nurs. 2007;15(1):40-7.

52. O'Kelly FD, Teljeur C, Carter I, Plunkett PK. Impact of a GP cooperative on lower acuity emergency department attendances. Emerg Med J. 2010;27(10):770-3.

53. Seaberg DC, Hennings J, Good M, et al. Redirecting low-acuity pediatric emergency department patients to a hospital-based federally qualified health center. Acad Emerg Med. 2010;17(Suppl. 1):S96.

54. Anonymous. ED navigators steer patients to appropriate providers. Hosp Case Manag. 2012;20(3):43-4.

55. Nguyen ND, Moore JB, McIntosh NP, Jones ML, Zimmerman J, Summers RL. Emergency department triage of low acuity patients to a Federally Qualified Health Center. J Miss State Med Assoc. 2013;54(10):280-3.

56. Bardelli P, Kaplan V. Non-urgent encounters in a Swiss medical emergency unit. Swiss Med Wkly. 2013;143(d10, 100970884):w13760.

57. Stone M, Nguygen ND, Moore JB, et al. Emergency department triage of low acuity patients to a federally qualified health center. Ann Emerg Med. 2013;62(4 Suppl. 1):S46-S7.

58. Ellbrant J, Akeson J, Akeson PK. Pediatric emergency department management benefits from appropriate early redirection of nonurgent visits. Pediatr Emerg Care. 2015;31(2):95-100.

59. Foster S, Bisset C. "prescribing the remedy: Co-located out-of-hours GP-what would this actually mean for a paediatric emergency department?". Arch Dis Child. 2015;100(Suppl. 3):A43-A4.

60. Blom MC, Erwander K, Gustafsson L, Landin-Olsson M, Jonsson F, Ivarsson K. Primary triage nurses do not divert patients away from the emergency department at times of high in-hospital bed occupancy - a retrospective cohort study. BMC Emerg Med. 2016;16(1):39.

61. Poropat F, Heinz P, Barbi E, Ventura A. Comparison of two European paediatric emergency departments: does primary care organisation influence emergency attendance? Ital J Pediatr. 2017;43(1):29.

62. Morin C, Choukroun J, Callahan J-C. Safety and efficiency of a redirection procedure toward an out of hours general practice before admission to an emergency department, an observational study. BMC Emerg Med. 2018;18(1):26.

63. Ali A, Karmani J. Audit of a newly developed ambulatory care service at Diana princess of Wales Hospital Grimsby UK. Postgrad Med J. 2018;94(Suppl. 1):A11.

64. Morton K, Voss S, Adamson J, et al. General practitioners and emergency departments (GPED)—efficient models of care: a mixed-methods study protocol. BMJ Open. 2018;8(10):e024012.

65. Brasseur E, Gilbert A, Petit M, Ghuysen A, D'Orio V. Advanced triage for self-referrals in the emergency department: The PERSEE algorithm. Crit Care. 2019;23(Suppl. 2).

66. McFadzean IJ, Edwards M, Davies F, et al. Realist analysis of whether emergency departments with primary care services generate 'provider-induced demand'. BMC Emerg Med. 2022;22(1):155.

67. Liedes-Kauppila M, Heikkinen AM, Rahkonen O, et al. Development of the use of primary health care emergency departments after interventions aimed at decreasing overcrowding: a longitudinal follow-up study. BMC Emerg Med. 2022;22(1):108.

68. Pincombe A, Schultz TJ, Hofmann D, Karnon J. Economic evaluation of a medical ambulatory care service using a single group interrupted time-series design. J Eval Clin Pract. 2023;29(2):329-40.

69. Di Nardo V, Caroli G, Del Grande A, Di Nardo L. In quale misura l'attuazione del modello See and Treat potrebbe contribuire alla gestione del sovraffollamento del pronto soccorso? Uno studio descrittivo retrospettivo. Scenario. 2023;40(2):20-4.

70. Cooper A, Edwards M, Davies F, et al. Programme theories to describe how different general practitioner service models work in different contexts in or alongside emergency departments (GP-ED): realist evaluation. Emerg Med J. 2024;41(5):287-95.

71. Dale J, Green J, Reid F, Glucksman E. Primary care in the accident and emergency department: I. Prospective identification of patients. BMJ (Clinical research ed). 1995;311(7002):423-6.

72. Feral-Pierssens A-L, Morris J, Marquis M, et al. Safety assessment of a redirection program using an electronic application for low-acuity patients visiting an emergency department. BMC Emerg Med. 2022;22(1):71.

73. Bentley JA, Thakore S, Morrison W, Wang W. Emergency Department redirection to primary care: a prospective evaluation of practice. Scott Med J. 2017;62(1):2-10.

74. Dias RD, Rios IC, Canhada CL, et al. Using the Manchester triage system for refusing nonurgent patients in the emergency department: A 30-day outcome study. J Emerg Manag. 2016;14(5):349-64.

75. Martin BC. Emergency medicine versus primary care: a case study of three prevalent, costly, and non-emergent diagnoses at a community teaching hospital. J Health Care Finance. 2000;27(2):51-65.

76. Lafay V, Giraud C, Bel C, Giovannetti O. [General practice consultation in a hospital emergency department. History, evaluation and prospects]. Presse Med. 2002;31(35):1643-9.

77. Fineberg DA, Stewart MM. Analysis of patient flow in the emergency room. Mount Sinai J Med. 1977;44(4):551-9.

78. Oslislo S, Witt K, von Stillfried D, et al. Zwischen Vision und Wirklichkeit: Untersuchung zur Machbarkeit der Weiterleitung von weniger dringlichen Hilfesuchenden in die ambulante Versorgung. Notf Rettungsmed. 2024.

79. Bermudez ES, Jones D, Gulden C, Moyer R, Horn J. The Right Care at the Right Place: Emergency Department to Same-Day Primary Care Referral Pilot. Acad Emerg Med. 2023;30(Suppl. 1):189-90.

80. Koech L, Strohl S, Lauerer M, et al. [Redirection of patients from the emergency department to ambulatory care: a feasibility study]. Gesundheitswesen. 2024;86(5):339-45.

Wrong patient population: Numbers 81-93

81. Koonar H, Kathy TK, Lang E, et al. An intergrated rapid assessment zone and waiting room care initiative improves throughput for CTAS-ITI and CTAS-IV patients. Can J Emerg Med. 2010;12(3):231.

82. Soremekun O, Shofer FS, Datner E, Moore J, Heidi K, Grasso D. The impact of an emergency department mid-track on patient flow. Ann Emerg Med. 2012;60(4 Suppl. 1):S106.

83. Colacone A, Guttman A, Afilalo M, et al. What is the impact of a rapid assessment zone on wait times to care for the acute care unit of the emergency department? Can J Emerg Med. 2012;14(Suppl. 1):S22.

84. Pothof J, Sharp B, Repplinger M, Schnepf J, Hamedani A. Flexible care area as an emergency department front-end solution. Ann Emerg Med. 2013;62(4 Suppl. 1):S12.

85. Arya R, Wei G, McCoy J, Ohman-Strickland PA. Decreasing turn-around-time with a split ESI 3 patient flow model. Acad Emerg Med. 2013;20(5 Suppl. 1):S41.

86. Soremekun OA, Shofer FS, Grasso D, Mills AM, Moore J, Datner EM. The effect of an emergency department dedicated midtrack area on patient flow. Acad Emerg Med. 2014;21(4):434-9.

87. Grouse AI, Bishop RO, Gerlach L, de Villecourt TL, Mallows JL. A stream for complex, ambulant patients reduces crowding in an emergency department. Emerg Med Australas. 2014;26(2):164-9.

88. Seaberg D, Elseroad S, Dumas M, et al. Patient Navigation for Patients Frequently Visiting the Emergency Department: A Randomized, Controlled Trial. Acad Emerg Med. 2017;24(11):1327-33.

89. Leung AK, Whatley SD, Gao D, Duic M. Impact of process improvements on measures of emergency department efficiency. Can J Emerg Med. 2017;19(2):96-105.

90. Garra G, Apterbach W, Gupta S. Split-flow model for managing abdominal pain: A before and after comparison of throughput metrics. Acad Emerg Med. 2020;27(Suppl. 1):S306-S7.

91. Muradian M, Pirkola M, Ziadeh J, Bork S. 14 The Impact of a Mixed Fast-Track and Mid-Acuity Track Area With a Vertical Component on Emergency Department Throughput. Ann Emerg Med. 2022;80(4 Suppl.):S7.

92. Arya R, Wei G, McCoy JV, Crane J, Ohman-Strickland P, Eisenstein RM. Decreasing length of stay in the emergency department with a split emergency severity index 3 patient flow model. Acad Emerg Med. 2013;20(11):1171-9.

93. Van der Linden MCC, Van Loon-van Gaalen MM, Meylaerts SAGS, et al. Improving emergency department flow by introducing four interventions simultaneously. A quality improvement project. Int Emerg Nurs. 2024;76:101499.

Wrong setting: Numbers 94-96

94. Fokkema CB, Janssen L, Roumen RMH, van Dijk WA. Optimizing the pathway for simple stable fractures. Bone Joint Open. 2023;4(10):728-34.

95. Bellantoni J, Tietz S. Implementation of an Acute Care Clinic to Reduce Low Acuity Emergency Department Use. J Am Geriatr Soc. 2023;71(Suppl. 1):S340.

96. Arain M, Nicholl J, Campbell M. GP-led walk-in centre in the UK: Another way for urgent healthcare provision. Crit Care. 2013;17(Suppl. 2):S97.

Other exclusion reasons (no fulltext available: Numbers 97-100, manually identified as duplicate in later screening stages: Numbers 101-115)

97. Longo M, Pavan A, Patriarca M, et al. [A new Emergency Department case management model for nonurgent patients]. Ig Sanita Pubbl. 2012;68(5):707-17.

98. Bloom R. Reorganizing ambulatory care: an administrator's view. J Ambul Care Manag. 1979;2(1):62-7.

99. Cooke J, Finneran K. A clearing in the crowd: innovations in emergency services. Paper series (United Hospital Fund of New York). 1994;(bsh, 8610109):1-43.

100. Bachofer HJ. An evaluation of the feasibility of hospital sponsored ambulatory care centers in Southwestern Oakland County. Abstr Hosp Manag Stud. 1977;14(2):17957-p.

101. Hampers LC, Cha S, Gutglass DJ, Binns HJ, Krug SE. Fast track and the pediatric emergency department: resource utilization and patients outcomes. Acad Emerg Med. 1999;6(11):1153-9.

102. Tsai V, Harley J, Sharieff G, Carlson LA, Kanegaye J. Rapid medical assessment: Improved patient flow and left without being seen rates. Pediatr Emerg Care. 2009;25(10):706.

103. Dinh M. Emergency department fast track: a randomised control trial of physician versus nurse-based care. Australian New Zealand Clinical Trials Registry. 2009:ACTRN12609000930280.

104. van Veen M, ten Wolde F, Poley MJ, Ruige M, van Meurs AHJ, Hable C, et al. Referral of nonurgent children from the emergency department to general practice: Compliance and cost savings. Eur J Emerg Med. 2011;19(1):14-9

105. Eichler K, Senn O, Ruthemann I, Bogli K, Sidler P, Brugger U. Reorganisation of hospital emergency services: A business case for quality improvement. Value Health. 2011;14(7):A344-A5.

106. Mackenzie RS, Burmeister DB, Brown JA, et al. Implementation of a rapid assessment unit (intake team): Impact on emergency department length of stay. Ann Emerg Med. 2013;62(4 Suppl. 1):S12-S3.

107. Arya R, Wei G, McCoy JV, et al. Decreasing Length of Stay in the Emergency Department With a Split Emergency Severity Index 3 Patient Flow Model. Acad Emerg Med. 2013;20(11):1171-9.

108. Soremekun OA, Shofer FS, Grasso D, et al. The Effect of an Emergency Department Dedicated Midtrack Area on Patient Flow. Acad Emerg Med. 2014;21(4):434-9.

109. Smith L, Narang Y, Edwardson K, et al. To GP or not to GP: Evaluation of children triaged to see a GP in a tertiary paediatric emergency department. Arch Dis Child. 2016;101(Suppl. 1):A115-A6.

110. Gasperini B, Fazi A, Maracchini G, Cherubini A. Is the emergency department fast-track system effective and safe for older patients? Eur Geriatr Med. 2018;9(Suppl. 1):S29.

111. Manice N, Greenwald P, Hsu H, et al. Comparison of 72-hour returns between telemedicine evaluation and traditional evaluation in the emergency department. Acad Emerg Med. 2018;25(Suppl. 1):S102.

112. Anderson JS, Burke RC, Augusto KD, et al. The Effect of a Rapid Assessment Zone on Emergency Department Operations and Throughput. Ann Emerg Med. 2019;75(2):236-245.

113. Doran KM, Colucci AC, Huang C, et al. Direct linkage of low-acuity emergency department patients with primary care: A pseudo-randomized controlled trial. Acad Emerg Med. 2012;19(Suppl. 1):S95.

114. Guttman A, Afilalo M, Colacone A, et al. What is the impact of a rapid assessment zone on wait times to care for the acute care unit of the emergency department? Acad Emerg Med. 2012;19(Suppl. 1):S197.

115. Brainard J, Rice A, Hughes G, Everden P. Service evaluation of "GP at Door" of Accident and Emergency Services in Eastern England. medRxiv. 2023;09.09:23295296.
