# Supplementary material for: Effectiveness and safety of emergency department-based streaming interventions for low-acuity utilizers - systematic review and meta-analysis
Source: BMC Emerg Med. 2026 Feb 19;26:58. doi: 10.1186/s12873-026-01488-w (PMC12922365; doi:10.1186/s12873-026-01488-w)
Supplement: Supplementary file 3 — Supplementary Material 3: Appendix 3 - Citations of included studies.pdf. Detailed list of included studies [file 12873_2026_1488_MOESM3_ESM.pdf]

Appendix 3: Citations of included studies

1. Abdullah Ghaleb WE, Almemari A, Qayyum H. 'See and Treat' Clinic Service Evaluation at a Tertiary Care Hospital in Abu Dhabi. Oman Med J. 2020;35(2):89-96.

2. Adriani L, Dall'oglio I, Brusco C, et al. Reduction of Waiting Times and Patients Leaving Without Being Seen in the Tertiary Pediatric Emergency Department: A Comparative Observational Study. Pediatr Emerg Care. 2022;38(5):219-23.

3. Agustin MS, Goldfrank L, Matz R, et al. Reorganization of ambulatory health care in an urban municipal hospital. Primary care and its impact on hospitalization. Arch Intern Med. 1976;136(11):1262-6.

4. Aksel G, Bildik F, Demircan A, et al. Effects of fast-track in a university emergency department through the national emergency department overcrowding study. J Pakistan Med Assoc. 2014;64(7):791-7.

5. Alabbasi K, Kruger E, Tennant M. Evaluation of Emergency Health-Care Initiatives to Reduce Overcrowding in a Referral Medical Complex, Jeddah, Saudi Arabia. Saudi J Health Syst Res. 2021;1(4):134-9.

6. AlDarrab A, Abuhaimed K, Alabdullah T, Alshabanah H, Almogbil M, Gletsu S. Impact of Triage physician and clinical operation management consultant implementation on emergency department throughput at a tertiary care center. Ann Emerg Med. 2009;54(3 Suppl. 1):S4-S5.

7. Aldus C, Pope I, Brainard J, Ruston A, Hughes G, Everden P. Feasibility and evaluation of an emergency department-based GP streaming and treatment service. medRxiv. 2022;05.13:22275043.

8. Aldus C, Pope I, Brainard J, Ruston A, Hughes G, Everden P. Feasibility and evaluation of an emergency department-based general practitioner streaming and treatment service. J Eval Clin Pract. 2023;29(3):485-94.

9. Anantharaman V. Impact of health care system interventions on emergency department utilization and overcrowding in Singapore. Int J Emerg Med. 2008;1(1):11-20.

10. Anderson JS, Burke RC, Augusto KD, et al. The Effect of a Rapid Assessment Zone on Emergency Department Operations and Throughput. Ann Emerg Med. 2020;75(2):236-45.

11. Ardagh MW, Wells JE, Cooper K, Lyons R, Patterson R, O'Donovan P. Effect of a rapid assessment clinic on the waiting time to be seen by a doctor and the time spent in the department, for patients presenting to an urban emergency department: a controlled prospective trial. N Z Med J. 2002;115(1157):U28.

12. Ashenburg N, Ribeira R, Lindquist B, Matheson LW, Shen S, Yiadom MYAB. Converting an ED Fast Track to an ED Virtual Visit Track. NEJM Catalyst. 2022;3(11):1-18.

13. Bellow Jr AA: A comparative analysis of two low-acuity flow processes in the Emergency Department. Dissertation thesis. Duquesne University. 2015.

14. Benger J, Brant H, Scantlebury A, Anderson H, Baxter H, Bloor K, et al. General practitioners working in or alongside the emergency department: the GPED mixed-methods study. Health Soc Care Deliv Res. 2022;10(30).

15. Bennage JK, Ford CD, Ezemenaka CJ, Persing TF. Emergency Department Length of Stay: A Community Hospital Initiative. Adv Emerg Nurs J. 2024;46(3):263-273.

16. Berkowitz DA, Brown K, Morrison S, et al. Improving Low-acuity Patient Flow in a Pediatric Emergency Department: A System Redesign. Pediatr Qual Saf. 2018;3(6):e122.

17. Bessert B, Oltrogge-Abiry JH, Peters PS, et al. Synergism of an Urgent Care Walk-in Clinic With an Emergency Department. Dtsch Arztebl Int. 2023;120(29-30):491-8.

18. Blaschke S, Dormann H, Somasundaram R, et al. OPTINOFA Study Group. Structured triage in the emergency department via intelligent assistant service OPTINOFA: Results of a multicenter, cluster-randomized and controlled interventional study in Germany. Med Klin Intensivmed Notfmed. 2025;120(7):585-595.

19. Boeke AJP, van Randwijck-Jacobze ME, de Lange-Klerk EM, Grol SM, Kramer MH, van der Horst HE. Effectiveness of GPs in accident and emergency departments. Br J Gen Pract. 2010;60(579):e378-84.

20. Bonalumi NM, Bhattacharya A, Edwards C, et al. Impact of a Planned Workflow Change: Super Track Improves Quality and Service for Low-Acuity Patients at an Inner-City Hospital. J Emerg Nurs. 2017;43(2):114-25.

21. Bond PA. A staffed ED assessment room: impact on wait times for nonurgent patients at a Saudi Arabian hospital. J Emerg Nurs. 2001;27(4):394-5.

22. Bosmans JE, Boeke AJ, van Randwijck-Jacobze ME, et al. Addition of a general practitioner to the accident and emergency department: A cost-effective innovation in emergency care. Emerg Med J. 2011;29(3):192-6.

23. Brainard J, Rice A, Hughes G, Everden P. Service evaluation of 'GP at Door' of accident and emergency services in Eastern England. Prim Health Care Res Dev. 2025 Jan 10;26:e5.

24. Broekman S, Van Gils-Van Rooij E, Meijboom B, De Bakker D, Yzermans C. Do out-of-hours general practitioner services and emergency departments cost more by collaborating or by working separately? A cost analysis. J Prim Health Care. 2017;9(3):212-9.

25. Celona CA, Amaranto A, Ferrer R, et al. Interdisciplinary Design to Improve Fast Track in the Emergency Department. Adv Emerg Nurs J. 2018;40(3):198-203.

26. Chartier L, Josephson T, Bates K, Kuipers M. Improving emergency department flow through Rapid Medical Evaluation unit. BMJ Qual Improv Rep. 2015;4(1):u206156.w2663.

27. Chmiel C, Wang M, Sidler P, Eichler K, Rosemann T, Senn O. Implementation of a hospital-integrated general practice--a successful way to reduce the burden of inappropriate emergency-department use. Swiss Med Wkly. 2016;146(d10, 100970884):w14284.

28. Chrusciel J, Fontaine X, Devillard A, et al. Impact of the implementation of a fast-track on emergency department length of stay and quality of care indicators in the Champagne-Ardenne region: a before-after study. BMJ Open. 2019;9(6):e026200.

29. Considine J, Kropman M, Kelly E, Winter C. Effect of emergency department fast track on emergency department length of stay: a case-control study. Emerg Med J. 2008;25(12):815-9.

30. Cooke MW, Wilson S, Pearson S. The effect of a separate stream for minor injuries on accident and emergency department waiting times. Emerg Med J. 2002;19(1):28-30.

31. Copeland J, Gray A. A Daytime Fast Track Improves Throughput in a Single Physician Coverage Emergency Department. Can J Emerg Med. 2015;17(6):648-55.

32. Dale J. Primary care in accident and emergency departments : the cost effectiveness and applicability of a new model of care. Dissertation thesis. University of London, London School of Hygiene and Tropical Medicine. 1998.

33. Dale J, Green J, Reid F, Glucksman E, Higgs R. Primary care in the accident and emergency department: II. Comparison of general practitioners and hospital doctors. BMJ (Clinical research ed.). 1995;311(7002):427-30.

34. Dale J, Lang H, Roberts JA, Green J, Glucksman E. Cost effectiveness of treating primary care patients in accident and emergency: a comparison between general practitioners, senior house officers, and registrars. BMJ (Clinical research ed). 1996;312(7042):1340-4.

35. Darrab AA, Fan J, Fernandes CMB, Zimmerman R, et al. How does fast track affect quality of care in the emergency department? Eur J Emerg Med. 2006;13(1):32-5.

36. Davies F, Edwards M, Price D, et al. Evaluation of different models of general practitioners working in or alongside emergency departments: a mixed-methods realist evaluation. Health Soc Care Deliv Res. 2024;12(10):1-152.

37. Davis F, Kwon N, Berman A, et al. Quick look process in the emergency department. Acad Emerg Med. 2020;27(Suppl. 1):S307.

38. Devkaran S, Parsons H, Van Dyke M, Drennan J, Rajah J. The impact of a fast track area on quality and effectiveness outcomes: a Middle Eastern emergency department perspective. BMC Emerg Med. 2009;9(100968543):11.

39. Dinh M, Walker A, Parameswaran A, Enright N. Evaluating the quality of care delivered by an emergency department fast track unit with both nurse practitioners and doctors. Aust Emerg Nurs J. 2012;15(4):188-94.

40. Doran KM, Colucci AC, Hessler RA, Ngai CK, Williams ND, Wallach AB, et al. An intervention connecting low-acuity emergency department patients with primary care: effect on future primary care linkage. Ann Emerg Med. 2013;61(3):312-21.e7.

41. Ducharme J, Alder RJ, Pelletier C, Murray D, Tepper J. The impact on patient flow after the integration of nurse practitioners and physician assistants in 6 Ontario emergency departments. Can J Emerg Med. 2009;11(5):455-61.

42. Eichler K, Hess S, Chmiel C, et al. Sustained health-economic effects after reorganisation of a Swiss hospital emergency centre: a cost comparison study. Emerg Med J. 2014;31(10):818-23.

43. Eller A. Rapid assessment and disposition: applying LEAN in the emergency department. J Healthc Qual. 2009;31(3):17-22.

44. Farion KJ, Tse S, Patrice J, et al. IMPACT of a tertiary pediatric emergency department ambulatory zone on wait times of both high and low acuity patients. Can J Emerg MEd. 2010;12(3):239.

45. Feral-Pierssens AL, Gaboury I, Carbonnier C, Breton M. Redirection of low-acuity emergency department patients to nearby medical clinics using an electronic medical support system: effects on emergency department performance indicators. BMC Emerg Med. 2024;24(1):166.

46. Fernandes CMB, Christenson JM, Price A. Continuous quality improvement reduces length of stay for fast-track patients in an emergency department. Acad Emerg Med. 1996;3(3):258-63.

47. Gadomski AM, Perkis V, Horton L, Cross S, Stanton B. Diverting managed care Medicaid patients from pediatric emergency department use. Pediatrics. 1995;95(2):170-8.

48. Gardner RM, Friedman NA, Carlson M, Bradham TS, Barrett TW. Impact of revised triage to improve throughput in an ED with limited traditional fast track population. Am J Emerg Med. 2018;36(1):124-7.

49. Gasperini B, Pierri F, Espinosa E, Fazi A, Maracchini G, Cherubini A. Is the fast-track process efficient and safe for older adults admitted to the emergency department? BMC Geriatr. 2020;20(1):154.

50. Gaughan J, Liu D, Gutacker N, Bloor K, Doran T, Benger JR. Does the presence of general practitioners in emergency departments affect quality and safety in English NHS hospitals? A retrospective observational study. BMJ Open. 2022;12(2):e055976.

51. Gibney D, Murphy AW, Barton D, et al. Randomized controlled trial of general practitioner versus usual medical care in a suburban accident and emergency department using an informal triage system. Br J Gen Pract. 1999;49(438):43-4.

52. Gils - van Rooij ESJ: The paradox of urgent care collaborations: a multi perspective study of cooperating emergency departments and general practitioners. Dissertation thesis. Tilburg University. 2016.

53. Gupta S, Willis H, Zeplin J, Kwon N, Amato TM. The low acuity tract: Innovative approach to evolving your "split flow" emergency department efficiency model. Acad Emerg Med. 2017;24(Suppl. 1):S202.

54. Hampers LC, Cha S, Gutglass DJ, Binns HJ, Krug SE. Fast track and the pediatric emergency department: Resource utilization and patient outcomes. Acad Emerg Med. 1999;6(11):1153-9.

55. Hansagi H, Allebeck P, Edhag O. Health care utilization after referral from a hospital emergency department. Scand J Soc Med. 1989;17(4):291-9.

56. Harris T, McDonald K. How do clinicians with different training backgrounds manage walk-in patients in the ED setting? Emerg Med J. 2014;31(12):975-9.

57. Hess S, Sidler P, Chmiel C, Bogli K, Senn O, Eichler K. Satisfaction of health professionals after implementation of a primary care hospital emergency centre in Switzerland: A prospective before-after study. Int Emerg Nurs. 2015;23(4):286-93.

58. Hsu H, Greenwald PW, Clark S, et al. Telemedicine Evaluations for Low-Acuity Patients Presenting to the Emergency Department: Implications for Safety and Patient Satisfaction. Telemed J E Health. 2020;26(8):1010-5.

59. Hussain A, LeBaron J. The Effect of Advanced Practice Provider Discharge on Patient Throughput in an Emergency Department Fast Track. Ann Emerg Med. 2020;76(4 Suppl.):S147.

60. Hwang CE, Lipman GS, Kane M. Effect of an emergency department fast track on Press-Ganey patient satisfaction scores. West J Emerg Med. 2015;16(1):34-8.

61. Ieraci S, Digiusto E, Sonntag P, Dann L, Fox D. Streaming by case complexity: evaluation of a model for emergency department Fast Track. Emerg Med Australas. 2008;20(3):241-9.

62. Jeanmonod R, Delcollo J, Jeanmonod D, Dombchewsky O, Reiter M. Comparison of resident and mid-level provider productivity and patient satisfaction in an emergency department fast track. Emerg Med J. 2013;30(1):e12.

63. Jiménez S, de la Red G, Miró Ò, et al. Efectividad de la incorporación de un médico especialista en medicina familiar y comunitaria en un servicio de urgencias hospitalario. Med Clin (Barc). 2005;125(4):132-7.

64. Joseph A, Patel A, Robinson RD, Andersen CA, Zenarosa NR, De Moor C, et al. The role of a split-flow model in the improvement of emergency department efficiency with respect to patients presenting with different acuity levels. Ann Emerg Med. 2013;62(4 Suppl. 1):S1-S2.

65. Kanzaria H, Mercer M, To J, et al. Using lean methodology to create a care pathway for low acuity emergency department patients in a safety net hospital. Acad Emerg Med. 2017;24(Suppl. 1):S202-S3.

66. Kelly AM, Bryant M, Cox L, Jolley D. Improving emergency department efficiency by patient streaming to outcomes-based teams. Aust Health Rev. 2007;31(1):16-21.

67. Kilic YA, Agalar FA, Kunt M, Cakmakci M. Prospective, double-blind, comparative fast-tracking trial in an academic emergency department during a period of limited resources. Eur J Emerg Med. 1998;5(4):403-6.

68. King DL, Ben-Tovim DI, Bassham J. Redesigning emergency department patient flows: application of Lean Thinking to health care. Emerg Med Australas. 2006;18(4):391-7.

69. Kool RB, Homberg DJ, Kamphuis HCM. Towards integration of general practitioner posts and accident and emergency departments: a case study of two integrated emergency posts in the Netherlands. BMC Health Serv Res. 2008;8(101088677):225.

70. Krakau I, Hassler E. Provision for clinic patients in the ED produces more nonemergency visits. Am J Emerg Med. 1999;17(1):18-20.

71. Kwa P, Blake D. Fast track: has it changed patient care in the emergency department? Emerg Med Australas. 2008;20(1):10-5.

72. Lam D, Braund C, Schmidt S, Johnson B, Spencer SP, Agbim C. How Super Is Supertrack? Expediting Care of Fast-track Patients through a Pediatric Emergency Department. Pediatr Qual Saf. 2024;9(5):e770.

73. Lee NK, Ahn YR, Kim YH, et al. Holiday fast-track reduced medical cost and length of emergency department stay: Preliminary report from a single secondary care hospital. Hong Kong J Emerg Med. 2015;22(2):84-92.

74. Lehto M, Mustonen K, Kantonen J, Raina M, Heikkinen A-MK, Kauppila T. A Primary Care Emergency Service Reduction Did Not Increase Office-Hour Service Use: A Longitudinal Follow-up Study. J Prim Care Community Health. 2019;10(101518419):2150132719865151.

75. Leigh S, Mehta B, Dummer L, et al. Management of non-urgent paediatric emergency department attendances by GPs: a retrospective observational study. Br J Gen Pract. 2021;71(702):e22-e30.

76. Liferidge A, McCarthy M, Blanchard J, Ding R, Li S. Triage of low acuity emergency department patients to a primary care clinic and medical home: A utilization and cost effectiveness analysis. Ann Emgerg Med. 2015;66(4 Suppl. 1):S87-S8.

77. Lo BM, Mendelson MH, Holder L, Berrios M, Kerr D. Building a super track: Use of lean to decrease turnaround times for low-acuity patients. Acad Emerg Med. 2013;20(5 Suppl. 1):S47.

78. Lydakis C, Patramanis J, Lavredaki K, Karavitaki M, Neofotistos G. Crowding in emergency departments: The role of a fast track clinic. Arch Hell Med. 2014;31(3):336-41.

79. MacKenzie RS, Burmeister DB, Brown JA, et al. Implementation of a rapid assessment unit (intake team): impact on ED length of stay. Am J Emerg Med. 2015;33(2):291-3.

80. Martin HA, Noble M, Wilmarth J. Improving Patient Flow and Decreasing Patient Length of Stay in the Pediatric Emergency Department Through Implementation of a Fast Track. Adv Emerg Nurs J. 2021;43(2):162-9.

81. McCarron M, Burgess K, Gibbs S. An evaluation of primary care streaming in a tertiary paediatric emergency department. Arch Dis Child. 2019;104(Suppl. 2):A138-A9.

82. McHugh C, Krinsky R, Sharma R. Innovations in Emergency Nursing: Transforming Emergency Care Through a Novel Nurse-Driven ED Telehealth Express Care Service. J Emerg Nurs. 2018;44(5):472-7.

83. Miro O, Salgado E, Tomas S, et al. [Direct discharge from triage in emergency departments: assessment, risks and patient satisfaction]. Med Clin (Barc). 2006;126(3):88-93.

84. Morreel S, Philips H, De Graeve D, et al. Triaging and referring in adjacent general and emergency departments (the TRIAGE trial): a cluster randomised controlled trial. PLoS One. 2021;16(11):e0258561.

85. Murphy AW, Plunkett PK, Bury G, et al. Effect of patients seeing a general practitioner in accident and emergency on their subsequent reattendance: cohort study. BMJ. 2000;320(7239):903-4.

86. Murphy AW, Bury G, Plunkett PK, et al. Randomised controlled trial of general practitioner versus usual medical care in an urban accident and emergency department: process, outcome, and comparative cost. BMJ (Clinical research ed). 1996;312(7039):1135-42.

87. Murrell KL, Offerman SR, Kauffman MB. Applying lean: implementation of a rapid triage and treatment system. West J Emerg Med. 2011;12(2):184-91.

88. O'Brien D, Williams A, Blondell K, Jelinek GA. Impact of streaming "fast track" emergency department patients. Aust Health Rev. 2006;30(4):525-32.

89. Penciner R, Ennis. Improving care of subacute patients in the emergency department: the Kaizen approach. Can J Emerg Med. 2008;10(3):1.

90. Perez B, Simon BC, English DK, Alter HJ, Hern HG. Novel approach to triage decreases left without being seen and average length of stay in lower acuity patients. Acad Emerg Med. 2010;17(Suppl. 1):S125-S6.

91. Pincombe A, Schultz TJ, Hofmann D, Karnon J. Economic evaluation of a medical ambulatory care service using a single group interrupted time-series design. J Eval Clin Pract. 2022;(cwd, 9609066).

92. Platter MEM, Kurvers RAJ, Janssen L, Verweij MMJ, Barten DG. The impact of an emergency care access point on pediatric attendances at the emergency department: An observational study. Am J Emerg Med. 2020;38(2):191-7.

93. Robinson M, Sampson A, Ticgelaor J. FastER care in the emergency department leads to improvement in emergency department throughput metrics as well as improved patient experience. Ann Emerg Med. 2016;68(4 Suppl. 1):S32-S3.

94. Rodi SW, Grau MV, Orsini CM. Evaluation of a fast track unit: alignment of resources and demand results in improved satisfaction and decreased length of stay for emergency department patients. Qual Manag Health Care. 2006;15(3):163-70.

95. Rogers T, Ross N, Spooner D. Evaluation of a 'See and Treat' pilot study introduced to an emergency department. Accid Emerg Nurs. 2004;12(1):24-7.

96. Ruocco D, Green JP, Sillero G, Berger T. Replacing traditional triage with a rapid evaluation unit decreases left-without-being-seen rate at a community emergency department. Acad Emerg Med. 2012;19(Suppl. 1):S26.

97. Saidi K, Paquet AL, Goulet H, et al. Effects of fast track implementation in an adult emergency department. Ann Fr Med Urgence. 2015;5(6):283-9.

98. Salisbury C, Hollinghurst S, Montgomery A, et al. The impact of co-located NHS walk-in centres on emergency departments. Emerg Med J. 2007;24(4):265-9.

99. Sanchez M, Smally AJ, Grant RJ, Jacobs LM. Effects of a fast-track area on emergency department performance. J Emerg Med. 2006;31(1):117-20.

100. Sayah A, Lai-Becker M, Kingsley-Rocker L, Scott-Long T, O'Connor K, Lobon LF. Emergency Department Expansion Versus Patient Flow Improvement: Impact on Patient Experience of Care. J Emerg Med 2016;50(2):339-48.

101. Scantlebury A, Adamson J, Salisbury C, et al. Do general practitioners working in or alongside the emergency department improve clinical outcomes or experience? A mixed-methods study. BMJ Open. 2022;12(9):e063495.

102. Scherer M, Boczor S, Weinberg J, Kaduszkiewicz H, Mayer-Runge U, Wagner H. Allgemeinmedizin in einer Universitätsklinik – Ergebnisse eines Pilotprojekts. Z Allg Med. 2014;90(4):9.

103. Seeger I, Rupp P, Naziyok T, Rölker-Denker L, Röhrig R, Hein A. Ambulante Versorgung in ZNA und Bereitschaftsdienstpraxis. Med Klin Intensivmed Notfmed. 2017;112(6):510-8.

104. Sharma A, Inder B. Impact of co-located general practitioner (GP) clinics and patient choice on duration of wait in the emergency department. Emerg Med J. 2011;28(8):658-61.

105. Shetty A, Gunja N, Byth K, Vukasovic M. Senior Streaming Assessment Further Evaluation after Triage zone: a novel model of care encompassing various emergency department throughput measures. Emerg Med Australas. 2012;24(4):374-82.

106. Short Apellaniz J, Álvaro de la Parra JA, Gomez-Meana A, et al. Leveraging Telemedicine to Reduce ED Overcrowding: The Quirónsalud Virtual Urgent Care Program. NEJM Catalyst. 2023;4(8):1-21.

107. Simon HK, Ledbetter DA, Wright J. Societal savings by "fast tracking" lower acuity patients in an urban pediatric emergency department. Am J Emerg Med. 1997;15(6):551-4.

108. Simon HK, McLario D, Daily R, Lanese C, Castillo J, Wright J. "Fast tracking" patients in an urban pediatric emergency department. Am J Emerg Med. 1996;14(3):242-4.

109. Smith L, Narang Y, Ibarz Pavon AB, et al. To GP or not to GP: a natural experiment in children triaged to see a GP in a tertiary paediatric emergency department (ED). BMJ Qual Saf. 2018;27(7):521-8.

110. Sukpraprut-Braaten S, Henderson JC, Kinchen DL, et al. Medical screening exam utilized to manage emergent and non-emergent patients presenting to the emergency department. Ann Emerg Med. 2016;68(4 Suppl. 1):S71.

111. Taylor K, Koonar H, Mercuur L, et al. An integrated intake and rapid assessment zone initiative improves throughput for lower-acuity patients. Acad Emerg Med. 2011;18(5 Suppl. 1):S182-S3.

112. Terris J, Leman P, O'Connor N, Wood R. Making an IMPACT on emergency department flow: improving patient processing assisted by consultant at triage. Emerg Med J. 2004:537-41.

113. Theunissen BHJJ, Lardenoye S, Hannemann PH, Gerritsen K, Brink PRG, Poeze M. Fast Track by physician assistants shortens waiting and turnaround times of trauma patients in an emergency department. Eur J Trauma Emerg Surg. 2014;40(1):87-91.

114. Thijssen WAMH, Kraaijvanger N, Barten DG, Boerma MLM, Giesen P, Wensing M. Impact of a well-developed primary care system on the length of stay in emergency departments in the Netherlands: a multicenter study. BMC Health Serv Res. 2016;16(101088677):149.

115. Thijssen WAMH, Wijnen-van Houts M, Koetsenruijter J, Giesen P, Wensing M. The impact on emergency department utilization and patient flows after integrating with a general practitioner cooperative: an observational study. Emerg Med Int. 2013;2013(101567070):364659.

116. Thompson B, Gross T, Mehalechko C, Conrad C, Wilson J. Split flow process in a pediatric emergency department. Acad Emerg Med. 2014;21(5 Suppl. 1):S104-S5.

117. Tsai VW, Sharieff GQ, Kanegaye JT, Carlson LA, Harley J. Rapid medical assessment: improving pediatric emergency department time to provider, length of stay, and left without being seen rates. Pediatr Emerg Care. 2012;28(4):354-6.

118. Uthman OA, Walker C, Lahiri S, et al. General practitioners providing non-urgent care in emergency department: a natural experiment. BMJ Open. 2018;8(5):e019736.

119. van der Baaren R, Barten DG, van Osch F, van Barneveld KWY, Janzing HMJ, Cals JWL. Minor traumatic injuries in the emergency department pre- and post-implementation of an emergency care access point. J Eval Clin Pract. 2022;(cwd, 9609066).

120. van der Heijden I, Schuwirth L. Changes in number and characteristics of patients attending the Accident and Emergency department after centralisation of GPs' out-of-hours care. Huisarts en Wetenschap. 2003;46(9):493-5.

121. van Gils-van Rooij ESJ, Yzermans CJ, Broekman SM, Meijboom BR, Welling GP, de Bakker DH. Out-of-Hours Care Collaboration between General Practitioners and Hospital Emergency Departments in the Netherlands. J Am Board Fam Med. 2015;28(6):807-15.

122. van Nuenen AB, de Jong D, Bongers F, et al. More emergency care, lower costs. Huisarts en Wetenschap. 2016;59(7):292-5.

123. van Uden CJT, Winkens RAG, Wesseling GJ, Crebolder HFJM, van Schayck CP. Use of out of hours services: a comparison between two organisations. Emerg Med J. 2003;20(2):184-7.

124. van Uden CJT, Nieman FHM, Voss GBWE, Wesseling G, Winkens RAG, Crebolder HFJM. General practitioners' satisfaction with and attitudes to out-of-hours services. BMC Health Serv Res. 2005;5(1):27. (van Uden 2005-1)

125. van Uden CJT, Winkens RAG, Wesseling G, Fiolet HFBM, van Schayck OCP, Crebolder HFJM. The impact of a primary care physician cooperative on the caseload of an emergency department: the Maastricht integrated out-of-hours service. J Gen Intern Med. 2005;20(7):612-7. (van Uden 2005-2)

126. van Uden CJT: Studies on general practice out-of-hours care. Dissertation thesis. Maastricht University. 2005. (van Uden 2005-3)

127. van Uden CJT, Ament AJHA, Voss GBWE, Wesseling G, Winkens RAG, van Schayck OCP, et al. Out-of-hours primary care. Implications of organisation on costs. BMC Fam Pract. 2006;7(100967792):29.

128. van Veelen MJ, van den Brand CL, Reijnen R, van der Linden MC. Effects of a general practitioner cooperative co-located with an emergency department on patient throughput. World J Emerg Med. 2016;7(4):270-3.

129. van Veen M, ten Wolde F, Poley MJ, et al. Referral of nonurgent children from the emergency department to general practice: compliance and cost savings. Eur J Emerg Med. 2012;19(1):14-9.

130. Verma A, Cheng I, Pardhan K, Notario L, Thomas-Boaz W, Shelton D. Using an ambulatory zone to improve physician initial assessment times in a tertiary care hospital emergency department. Can J Emerg Med. 2020;22(Suppl. 1):S21.

131. Vinton D, Riordan JP, O'Conner R, Joseph JW. Impact of the management of low-acuity patients at triage on their length of stay. Acad Emerg Med. 2019;26(Suppl. 1):S235-S6.

132. Wackers EME, Stadhouders NW, Maessen MFH, et al. Association between acute care collaborations and health care utilization as compared to stand-alone facilities in the Netherlands: a quasi-experimental study. Eur J Emerg Med. 2023;30(1):15-20

133. Wang M, Wild S, Hilfiker G, et al. Hospital-integrated general practice: a promising way to manage walk-in patients in emergency departments. J Eval Clin Pract. 2014;20(1):20-6.

134. Ward P, Huddy J, Hargreaves S, Touquet R, Hurley J, Fothergill J. Primary care in London: an evaluation of general practitioners working in an inner city accident and emergency department. J Accid Emerg Med. 1996;13(1):11-5.

135. Wiederhold BD, Perez B, Simon B. Novel approach to triage and patient flow decreases time to provider and LOS in lower-acuity patients. Acad Emerg Med. 2011;18(5 Suppl. 1):S182.

136. Yau FF-F, Tsai T-C, Lin Y-R, Wu K-H, Syue Y-J, Li C-J. Can different physicians providing urgent and non-urgent treatment improve patient flow in emergency department? The Am J Emerg Med. 2018;36(6):993-7.

137. Zaboli A, Brigo F, Garbin T, et al. Fast-track and primary care in the emergency department: managing more, but not decongesting. Intern Emerg Med. 2025, Epub ahead of print.
