# Supplementary material for: Effectiveness and safety of emergency department-based streaming interventions for low-acuity utilizers - systematic review and meta-analysis
Source: BMC Emerg Med. 2026 Feb 19;26:58. doi: 10.1186/s12873-026-01488-w (PMC12922365; doi:10.1186/s12873-026-01488-w)
Supplement: Supplementary file 4 — Supplementary Material 4: Appendix 4 - Study characteristics and certainty of evidence tables.pdf. Tables detailing study characteristics and GRADE rating of evidence certainty. Captions for the tables in Appendix 4. Table 1: Characteristics of included studies. Table 2: Rating of evidence certainty (modified GRADE summary of findings table) [file 12873_2026_1488_MOESM4_ESM.pdf]

Appendix 4: Study characteristics and certainty of evidence tables

Table 1: Characteristics of included studies

Author	Year	Country	Streaming	Subtype / Streaming location	Design	Time frame [a]	Special intervention components [b]	Control [c]	Intervention group(s) / after period(s) [d]	Control group / before period [d]	Popul. (General / LA only)
**GP streaming interventions: internal**											
Bessert	2023	Germany	GP	Internal	BA	2	1	SC	2376	2389	LA
Boeke [p]	2010	Netherlands	GP	Internal	BA	3		SC	695	832	LA
Bosch-van Nuenen	2016	Netherlands	GP	Internal	BA	4	1	SC	22318	16969	General
Bosmans [p]	2011	Netherlands	GP	Internal	BA	3		SC	695	832	LA
Brainard	2024	UK	GP	Internal	Parallel, P	16	3	SC	28868	N/D	LA
Broekman [q]	2017	Netherlands	GP	Internal	Parallel, R	9	1	Separate operation of GP and ED	58620	63441	General
Chmiel [r]	2016	Switzerland	GP	Internal	BA	Multiple		SC	1151;559;1631	1055	LA
Dale [s]	1995	UK	GP	Internal	Parallel, CCT, P	12		Treatment by ED staff	1702;557	2382	LA
Dale [s]	1996	UK	GP	Internal	Parallel, CCT, P	12		Treatment by ED staff	1702;557	2382	LA
Dale [s]	1997	UK	GP	Internal	Parallel, CCT, P	12		Treatment by ED staff	1702;557	2382	LA
Eichler [r]	2014	Switzerland	GP	Internal	BA	Multiple		SC	690;533;1095	583	LA
Gibney	1999	Ireland	GP	Internal	Parallel, CCT, P	7		SC	771	1107	LA
Harris	2014	UK	GP	Internal	Parallel, P	1		SC	250	134	LA
Hess [r]	2015	Switzerland	GP	Internal	BA	N/D		SC	18;22	20	Medical staff
Jimenez	2005	Spain	GP	Internal	BA	1		SC	100	100	General
Kool	2008	Netherlands	GP	Internal	Combination, CA	N/D		SC	18255	17040	General

Author	Year	Country	Streaming	Subtype / Streaming location	Design	Time frame [a]	Special intervention components [b]	Control [c]	Intervention group(s) / after period(s) [d]	Control group / before period [d]	Popul. (General / LA only)
Krakau	1999	Sweden	GP	Internal	BA	19	1	SC	N/D [f]	N/D [f]	General
Leigh [t]	2021	UK	GP	Internal	Parallel, R	24		SC	8404	4695	LA
McCarron	2019	UK	GP	Internal	Parallel, R	12		SC	1094	2552	LA
Murphy [u]	1996	Ireland	GP	Internal	Parallel, CCT, P	15		SC	2303	2381	LA
Murphy [u]	2000	Ireland	GP	Internal	Parallel, CCT, P	16		SC	2209	2263	LA
Platter	2019	Netherlands	GP	Internal	BA	12	1	SC	716	888	General
Scherer	2014	Germany	GP	Internal	BA	3 (d)		SC	263	1621	LA
Smith [t]	2017	UK	GP	Internal	Parallel, R	6	1	SC	2821	2402	LA
Thijssen	2013	Netherlands	GP	Internal	BA, ITS	35	1	SC	102101	103372	General
Thijssen	2016	Netherlands	GP	Internal	Parallel, R	N/D	1	SC	4000	3000	General
van der Baaren	2022	Netherlands	GP	Internal	BA	12	1	SC	1181	1591	LA
van der Heijden	2003	Netherlands	GP	Internal	BA	0.5	1	SC	2694	1646	General
van Gils-van Rooij [q]	2015	Netherlands	GP	Internal	Parallel, R	9	1	Separate operation of GP and ED	58620	63441	General
van Rooij [q]	2016	Netherlands	GP	Internal	Parallel, R	4	1	SC	58620	63441	General
van Uden 2005-2 [v]	2005	Netherlands	GP	Internal	BA	0.7	1	SC	2278	2199	General
Wackers	2023	Netherlands	GP	Internal	Parallel, R	12	1	SC	466415	144430	General
Wang [r]	2014	Switzerland	GP	Internal	BA	1.3 (d)		SC	342	451	LA
Ward	1996	UK	GP	Internal	Parallel, P	1.3		SC	566	404	LA
**GP streaming interventions: co-located**											
Agustin	1976	US	GP	Co-located	BA	18		SC	351956	301473	General
Alabbasi	2021	Saudi Arabia	GP	Co-located	BA	12 (d)		SC	77139	156859	General
Aldus [w]	2022	UK	GP	Co-located	BA	2.5 (d)		SC	769	N/D [g]	LA
Aldus [w]	2023	UK	GP	Co-located	BA	2.5 (d)		SC	769	N/D [g]	LA

Author	Year	Country	Streaming	Subtype / Streaming location	Design	Time frame [a]	Special intervention components [b]	Control [c]	Intervention group(s) / after period(s) [d]	Control group / before period [d]	Popul. (General / LA only)
Blaschke	2025	Germany	GP	Co-located	BA, SWCR	11.5	1, 8	SC	26338	32364	General
Doran	2013	US	GP	Co-located	Parallel, CCT, P	12		SC	662;112	191	LA
Liferidge	2015	US	GP	Co-located	BA	12		SC	N/D	N/D [g]	General
Morreel	2021	Belgium	GP	Co-located	Parallel, CCT, P	10	1	SC	6374	1784	General
Seeger	2017	Germany	GP	Co-located	BA	1	1	SC	164	160	General
Sharma	2010	Australia	GP	Co-located	Parallel, R	12		SC	N/D [h]	N/D [h]	General
Uthman	2018	UK	GP	Co-located	Parallel, R	11	1	SC	5426	10852	LA
van Veelen	2016	Netherlands	GP	Co-located	BA	2	1	SC	8311	7936	General
**GP streaming interventions: external**											
Feral-Pierssens	2024	Canada	GP	External	BA, ITS	24	1	SC	121116	121856	General
Hansagi	1989	Sweden	GP	External	Combination, CA	13	1	SC	189	107	LA
Lehto	2019	Finland	GP	External	BA, ITS	84		SC	N/D [f]	N/D [f]	General
van Veen	2012	Netherlands	GP	External	BA	8 (d)	1	SC	247	1946	LA
**GP streaming interventions: combinations of locations**											
van Uden [v]	2003	Netherlands	GP	Comb.: Internal, external	Parallel, P	0.75	1	Separate operation of GP and ED	3054	3825	General
van Uden 2005-1 [v]	2005	Netherlands	GP	Comb.: Internal, external	Parallel, P	4	1	Separate operation of GP and ED	50	50	Medical staff
van Uden 2005-3 [v]	2005	Netherlands	GP	Comb.: Internal, external	BA [e]	2	1	SC	12319	11781	General

Author	Year	Country	Streaming	Subtype / Streaming location	Design	Time frame [a]	Special intervention components [b]	Control [c]	Intervention group(s) / after period(s) [d]	Control group / before period [d]	Popul. (General / LA only)
van Uden [v]	2006	Netherlands	GP	Comb.: Internal, external	Parallel, R	12	1	Separate operation of GP and ED	190000	285000	LA
Benger [x]	2022	UK	GP	Comb.: Internal, co-located	Parallel, R	12		Times with GP track not operational	~4.3 million	N/D [i]	General
Davies	2024	UK	GP	Comb.: Internal, co-located	Combination, ITS	96		SC	2426062 [j]	1558841	General
Gaughan [x]	2022	UK	GP	Comb.: Internal, co-located	Parallel, R	12		SC	4441349	N/D [i]	General
Scantlebury [x]	2022	UK	GP	Comb.: Internal, co-located	Parallel, R	12		SC	N/D	N/D	General
**ED streaming interventions**											
Aksel	2014	Turkey	ED		BA	0.25	2	SC	249	239	General
Al Darrab	2006	Canada	ED		BA	0.25	2	SC	468	465	LA
AlDarrab	2009	Saudi Arabia	ED		BA	6	4	SC	24314	22987	General
Anderson	2019	US	ED		BA	6	3, 5	ED fast track	44792	43847	General
Ardagh	2002	New Zealand	ED		Parallel, CCT, P	2.5	3	SC	2263	2204	General
Ashenburg	2022	US	ED		Parallel, R	N/D	7	ED fast track	2232	N/D [m]	LA
Bellow	2015	US	ED		Parallel, R	3	2	Rapid assessment	4542	3295	General
Bennage	2024	US	ED		BA	1	2, 3, 4, 5	SC	2211	2361	General

Author	Year	Country	Streaming	Subtype / Streaming location	Design	Time frame [a]	Special intervention components [b]	Control [c]	Intervention group(s) / after period(s) [d]	Control group / before period [d]	Popul. (General / LA only)
Berkowitz	2018	US	ED		BA	3.5 (d)	5	SC	N/D [k]	N/D	General
Bonalumi	2016	US	ED		BA	4 (d)	2, 3	SC	N/D [k]	N/D	LA
Bond	2001	Saudi Arabia	ED		BA	N/D	3	SC	200	200	LA
Celona	2018	US	ED		BA	N/D	2, 6	Rapid assessment	21053	15676	LA
Chartier	2015	Canada	ED		BA	Multiple	2	SC	1199;1650	1482	General
Chrusciel	2019	France	ED		BA	12	2	SC	57965	53768	General
Considine	2008	Australia	ED		BA	3 (d)	2	SC	822	822	General
Cooke	2002	UK	ED		BA	1.25	2	SC	6801	7117	General
Copeland	2015	Canada	ED		BA	12	2	SC	3729	3575	General
Davis	2020	US	ED		Parallel, R	N/D	3	Split-flow intake area	N/D [k]	N/D	General
Devkaran	2009	United Arab Emirates	ED		BA	1	2	SC	5706	4779	General
Dinh	2012	Australia	ED		Parallel, RCT	12	2, 6	SC	165	155	General
Ducharme	2009	Canada	ED		Parallel, R	7.5	6	SC	694	9313	General
Eller	2009	US	ED		BA, ITS	18 (d)	3	SC	N/D [f]	N/D [f]	General
Farion	2010	Canada	ED		BA	6	3	SC	N/D [k]	N/D	General
Fernandes	1996	Canada	ED		BA	Multiple	2, 5	SC	170;198;206	217	General
Gardner	2018	US	ED		BA	0.5	5, 6	SC	120	5328	LA
Gasperini	2020	Italy	ED		BA	12	2	SC	504	504	LA
Ghaleb	2020	United Arab Emirates	ED		Parallel, R	13	2	SC	11329;6238	25452	LA
Gupta	2017	US	ED		BA	N/D	2, 3	SC	N/D [f,k]	N/D	General
Hampers	1999	US	ED		Parallel, P	3	2	SC	479	557	LA
Hsu	2020	US	ED		Parallel, R	14.5	7	SC	3266	21129	LA
Hussain	2020	US	ED		BA	1	2, 6	SC	2291	1960	General
Hwang	2015	US	ED		BA	5	2	SC	85	140	General
Ieraci	2008	Australia	ED		Combination	6	2, 5	SC	6062	12442	General
Jeanmonod	2012	US	ED		Parallel, R	5	2, 6	SC	N/D [f,k]	N/D	LA
Joseph	2013	US	ED		BA, IST	7	2	SC	N/D [f,l]	N/D	General

Author	Year	Country	Streaming	Subtype / Streaming location	Design	Time frame [a]	Special intervention components [b]	Control [c]	Intervention group(s) / after period(s) [d]	Control group / before period [d]	Popul. (General / LA only)
Kanzaria	2017	US	ED		BA	N/D	2	SC	N/D	N/D	General
Kelly	2007	Australia	ED		BA	12	2	SC	31515	31570	General
Kilic	1998	Turkey	ED		Parallel, CCT, P	1	2	SC	143	126	LA
King	2006	Australia	ED		BA	12	2	SC	50337	49075	General
Kwa	2008	Australia	ED		BA	6	2	SC	20460	18267	General
Lam	2024	US	ED		BA	10 (d)	2	SC	N/D	N/D	LA
Lee	2015	South Korea	ED		BA	2	2	SC	1237	1116	General
Lo	2013	US	ED		BA	10	2	SC	11640	12880	LA
Lydakis	2014	Greece	ED		BA	5	2	SC	52697	45688	General
Mackenzie	2015	US	ED		BA	N/D	3	SC	10776	11911	General
Martin	2021	US	ED		Parallel, R	14	2	SC	N/D [k]	N/D	General
McHugh	2018	US	ED		Parallel, R	5	7	SC	1850	70000	General
Murrell	2011	US	ED		BA	6	2, 3, 5	SC	33926	30981	General
O'Brien	2006	Australia	ED		BA	3	2	SC	N/D [f,k]	N/D	General
Penciner	2008	Canada	ED		BA	4	2, 3, 4, 5	SC	7102	5203	LA
Perez	2010	US	ED		BA	1	2, 3, 4	SC	N/D [k]	N/D	General
Robinson	2016	US	ED		BA	4	2	SC	16511	N/D	General
Rodi	2006	US	ED		BA	1	2, 3, 4, 5, 6	SC	91	87	LA
Rogers	2004	UK	ED		BA	0.75	2, 4, 5, 6	SC	N/D [k]	N/D	LA
Ruocco	2012	US	ED		BA	10	3	SC	N/D [f,k]	N/D [f]	General
Saidi	2015	France	ED		BA	12	2, 4, 5	SC	59844	57444	General
Sanchez	2006	US	ED		BA	12	2, 6	SC	N/D [f,k]	N/D [f]	General
Sayah	2016	US	ED		BA, ITS	Multiple	3, 4, 5	SC	N/D [f,k]	N/D [f]	General
Shetty	2012	Australia	ED		BA	2.56	2, 3, 4, 5	SC	10713	10185	General
Short Apellaniz	2023	Spain	ED		Parallel, R	10	7	SC	17697	484080	General
Simon [y]	1996	US	ED		Parallel, R	9	2	SC	2243	N/D	General
Simon [y]	1997	US	ED		Parallel, R	12	2	SC	4060	5199	General
Taylor	2011	Canada	ED		BA	5	2, 3, 5	SC	N/D	N/D	LA
Terris	2004	UK	ED		Parallel, CCT, P	3	3	SC periods	378	N/D	General

Author	Year	Country	Streaming	Subtype / Streaming location	Design	Time frame [a]	Special intervention components [b]	Control [c]	Intervention group(s) / after period(s) [d]	Control group / before period [d]	Popul. (General / LA only)
Theunissen	2014	Netherlands	ED		BA	3	2, 4, 6	SC	1378	1280	General
Thompson	2014	US	ED		BA	6	3	SC	N/D [f,k]	N/D [f]	General
Tsai	2012	US	ED		BA	6	3	SC	32053	28360	General
Verma	2020	Canada	ED		Parallel, P	N/D	2, 3, 5	SC	N/D	N/D	General
Vinton	2019	US	ED		BA	1.3	2, 3	SC	556	562	General
Wiederhold	2011	US	ED		BA	2	2, 3, 4, 5, 6	SC	N/D [f]	N/D [f]	General
Yau	2017	Taiwan	ED		Parallel, R	12	4	All urgencies treated by same personnel	21630	15950	General
**UC streaming interventions**											
Pincombe	2022	Australia	UC	Co-located	BA, ITS	18		SC	553	546	LA
Salisbury	2007	UK	UC	Co-located	Combination, CA	N/D		SC	1546	1530	General
**Combinations of streaming interventions**											
Anantharaman	2008	Singapore	Comb.: GP, UC	Comb.: Co-located, external	BA	Multiple		SC	N/D [n]	N/D [n]	General
Gadomski	1995	US	Comb.: GP, UC	Comb.: Co-located, external	BA	6	1	Before gatekeeping	8399	8798	General
Sukpraprut-Braaten	2016	US	Comb.: GP, UC	Comb.: Co-located, external	BA	N/D		SC	N/D [o]	N/D	General
Miro	2006	Spain	Comb.: GP, UC	External	Parallel, CCT, P	12		Referral to UC center	25741	19023	General

Author	Year	Country	Streaming	Subtype / Streaming location	Design	Time frame [a]	Special intervention components [b]	Control [c]	Intervention group(s) / after period(s) [d]	Control group / before period [d]	Popul. (General / LA only)
Adriani	2022	Italy	Comb.: ED, UC	Co-located	BA	3	2	SC	15585	14822	General
Zaboli	2025	Italy	Comb.: GP, ED	Internal	Parallel, R	26	2	SC	20795;26351	120357	LA

RCT = randomized controlled trial; CCT = controlled clinical trial; CA = cohort analytic; ITS = interrupted time series; P = parallel prospective design; R = parallel retrospective design; SWCT = stepped-wedge cluster randomized trial; N/D = not determined, e.g. in case of insufficient data to unequivocally determine respective characteristics, or if several different values or data points were reported; SC = standard ED care; LA = low acuity; Popul. = Population.

[a] Timeframe = Length of parallel investigation or of periods compared (BA), in months. If length of before and after periods for BA studies different, length of after period is reported in table and marked with “(d)”. Also marked if multiple after time periods were analyzed/compared.

[b] Special intervention components: 1 = cooperation with external providers, 2 = fast track, 3 = rapid assessment/triage, 4 = different assignments of ED personnel, 5 = modification of care environment, 6 = treatment by non-physician staff, 7 = telemedicine, 8 = algorithm-guided treatment allocation

[c] Control = care provided in control group (parallel studies) or before period (BA studies).

[d] Participant numbers. For trials with more than one intervention or after period, n for further groups/periods are additionally listed, separated by semicolon(s).

[e] Thesis contains data also reported in other included papers. Information in this study characteristics table exclusively refers to an additional module not reported in the other included publications by the author.

[f] Patient volumes or (average) visits per time interval reported.

[g] Data for patient numbers treated and not treated in trial intervention available, but no population sizes for BA comparison reported.

[h] Total patient number reported (n = 1158474 visits analyzed) but not specified into intervention and control.

[i] Different interventions compared, only total n for population reported.

[j] Data refers to post-intervention patient number. Pre-intervention: n = 2520174 in intervention sites.

[k] General census data stated for study setting(s).

[l] Total patient number reported (n = 162901 visits analyzed) but not specified into before and after periods.

[m] Declared as “matched cohort” without actual reporting of population size.

[n] Multiple time periods and interventions reported with respective population sizes.

[o] Total patient number reported (n = 158620 visits analyzed) but not specified into before and after periods.

[p,q,r,s,t,u,v,w,x,y] related publications reporting on a common research project.

Table 2: Rating of evidence certainty (modified GRADE summary of findings table)

Certainty assessment							Effect		Certainty [f]
№ of studies, total n	Study design	Risk of bias [a]	Inconsistency [b]	Indirectness [c]	Imprecision	Other considerations	Relative (95% CI)	Absolute (95% CI)	
**POTENTIAL FOR ALTERNATIVE CARE**									
**Unselected patients, GP streaming**									
14 studies, 278.968 patients	13 non-randomized studies, 1 SWCT	very serious	very serious	serious	not serious	none	Proportion **0.32** (0.17 to 0.51)	-	⨁◯◯◯ Very low
**Low-acuity patients, GP streaming**									
11 studies, 58.175 patients	10 non-randomized studies, 1 SWCT	very serious	very serious	serious	not serious	none	Proportion **0.49** (0.34 to 0.64)	-	⨁◯◯◯ Very low
**Unselected patients, ED streaming**									
15 studies, 680.320 patients	non-randomized studies	very serious	very serious	serious	not serious	none	Proportion **0.25** (0.15 to 0.37)	-	⨁◯◯◯ Very low
**Low-acuity patients, ED streaming**									
9 studies, 95.420 patients	non-randomized studies	very serious	very serious	serious	not serious	none	Proportion **0.30** (0.14 to 0.51)	-	⨁◯◯◯ Very low
**WAITING TIME**									
**Unselected patients, GP streaming**									
3 studies, 301.290 patients	2 non-randomized studies, 1 SWCT	not serious	very serious	not serious	not serious	none	-	SMD **0.15 lower** (0.20 lower to 0.09 lower)	⨁◯◯◯ Very low

Certainty assessment							Effect		Certainty [f]
№ of studies, total n	Study design	Risk of bias [a]	Inconsistency [b]	Indirectness [c]	Imprecision	Other considerations	Relative (95% CI)	Absolute (95% CI)	
**Low-acuity patients, GP streaming**									
2 studies, 993 patients	non-randomized studies	very serious	very serious	not serious	not serious	none	-	SMD **0.06 lower** (0.47 lower to 0.36 higher)	⨁◯◯◯ Very low
**Unselected patients, ED streaming**									
10 studies, 367.005 patients	9 non-randomized studies, 1 parallel design RCT	very serious	very serious	not serious	not serious	none	-	SMD **0.41 lower** (0.68 lower to 0.14 lower)	⨁◯◯◯ Very low
**Low-acuity patients, ED streaming**									
5 studies, 12.323 patients	non-randomized studies	very serious	very serious	not serious	not serious	strong association [d]	-	SMD **0.83 lower** (1.26 lower to 0.4 lower)	⨁◯◯◯ Very low
**High-acuity patients, ED streaming**									
2 studies, 7.906 patients	non-randomized studies	very serious	very serious	not serious	not serious	none	-	SMD **0.57 lower** (1.41 lower to 0.26 higher)	⨁◯◯◯ Very low
**LENGTH OF STAY**									
**Unselected patients, GP streaming**									
2 studies, 272.804 patients	1 non-randomized study, 1 SWCT	not serious	serious	not serious	not serious	none	-	SMD **0.07 lower** (0.09 lower to 0.06 lower)	⨁◯◯◯ Very low
**Low-acuity patients, GP streaming**									

Certainty assessment							Effect		Certainty [f]
№ of studies, total n	Study design	Risk of bias [a]	Inconsistency [b]	Indirectness [c]	Imprecision	Other considerations	Relative (95% CI)	Absolute (95% CI)	
4 studies, 19.315 patients	non-randomized studies	serious	very serious	not serious	not serious	strong association [d]	-	SMD **0.85 lower** (1.37 lower to 0.33 lower)	⨁◯◯◯ Very low
**Unselected patients, ED streaming**									
11 studies, 505.933 patients	non-randomized studies	very serious	very serious	not serious	not serious	none	-	SMD **0.25 lower** (0.34 lower to 0.16 lower)	⨁◯◯◯ Very low
**Low-acuity patients, ED streaming**									
12 studies, 155.931 patients	non-randomized studies	very serious	very serious	not serious	not serious	none	-	SMD **0.39 lower** (0.56 lower to 0.22 lower)	⨁◯◯◯ Very low
**High-acuity patients, ED streaming**									
5 studies, 65.610 patients	non-randomized studies	very serious	very serious	not serious	not serious	none	-	SMD **0.1 lower** (0.22 lower to 0.02 higher)	⨁◯◯◯ Very low
**HOSPITAL ADMISSIONS**									
**GP streaming**									
18 studies, 4.960.574 patients [e]	non-randomized studies	very serious	very serious	not serious	not serious	none	**OR 0.72** (0.52 to 0.99)	-	⨁◯◯◯ Very low
**ED streaming**									
13 studies, 482.250 patients	12 non-randomized studies, 1 parallel design RCT	very serious	very serious	not serious	not serious	none	**OR 0.69** (0.45 to 1.04)	-	⨁◯◯◯ Very low

Certainty assessment							Effect		Certainty [f]
№ of studies, total n	Study design	Risk of bias [a]	Inconsistency [b]	Indirectness [c]	Imprecision	Other considerations	Relative (95% CI)	Absolute (95% CI)	
**LEAVING WITHOUT BEING SEEN**									
**GP streaming**									
7 studies, 283.066 patients [e]	non-randomized studies	serious	very serious	not serious	not serious	none	**OR 0.53** (0.31 to 0.93)	-	⨁◯◯◯ Very low
**ED streaming**									
15 studies, 609.443 patients	non-randomized studies	very serious	very serious	not serious	not serious	none	**OR 0.45** (0.31 to 0.66)	-	⨁◯◯◯ Very low
**UNPLANNED ED REATTENDANCES**									
**GP streaming**									
6 studies, 34.102 patients [e]	5 non-randomized studies, 1 SWCT	serious	not serious	not serious	not serious	none	**OR 0.97** (0.94 to 1.00)	-	⨁◯◯◯ Very low
**ED streaming**									
7 studies, 197.424 patients	non-randomized studies	very serious	very serious	not serious	not serious	none	**OR 0.83** (0.52 to 1.34)	-	⨁◯◯◯ Very low

RCT = randomized controlled trial; SWCT = stepped-wedge cluster randomized trial.

[a] Risk of bias: if "weak" studies were included in the respective meta-analysis, risk of bias (ROB) was judged as "very serious" in case of ≥30% of studies rated as "weak", otherwise as "serious". If only "strong" and "moderate" studies were included, ROB was judged as "serious" if ≥30% of studies were rated as "moderate".

[b] Inconsistency was rated as "very serious" if there was substantial between-study heterogeneity, as "serious" in case of moderate heterogeneity (as indicated by I^2 / prediction intervals).

[c] For potential for alternative care, indirectness was rated as "serious", as this was not assessed as an outcome in the studies, but derived from shares treated in one condition vs. the other.

[d] A strong association was indicated by an SMD of >0.8.

[e] Several meta-analyses for GP streaming include odds ratios from studies in which population size cannot be exactly determined; thus these are not included in total n reported in the table (two studies for hospital admissions; one study for leaving without being seen and unplanned ED reattendances, respectively).

[f] Certainty of the evidence calculated by GRADEpro GDT from domains.
