# Supplementary material for: Effectiveness and safety of emergency department-based streaming interventions for low-acuity utilizers - systematic review and meta-analysis
Source: BMC Emerg Med. 2026 Feb 19;26:58. doi: 10.1186/s12873-026-01488-w (PMC12922365; doi:10.1186/s12873-026-01488-w)

Appendix 5: Risk of bias (Effective Public Health Practice Project tool ratings)

Figure 7: Risk of bias in GP streaming studies

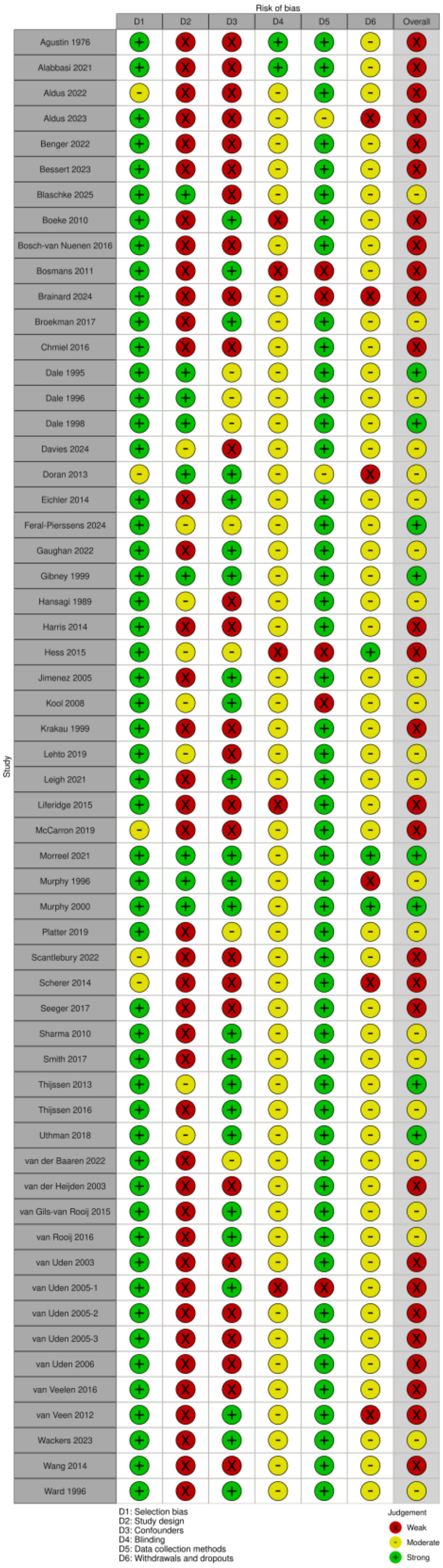

Figure 8: Risk of bias in ED streaming studies

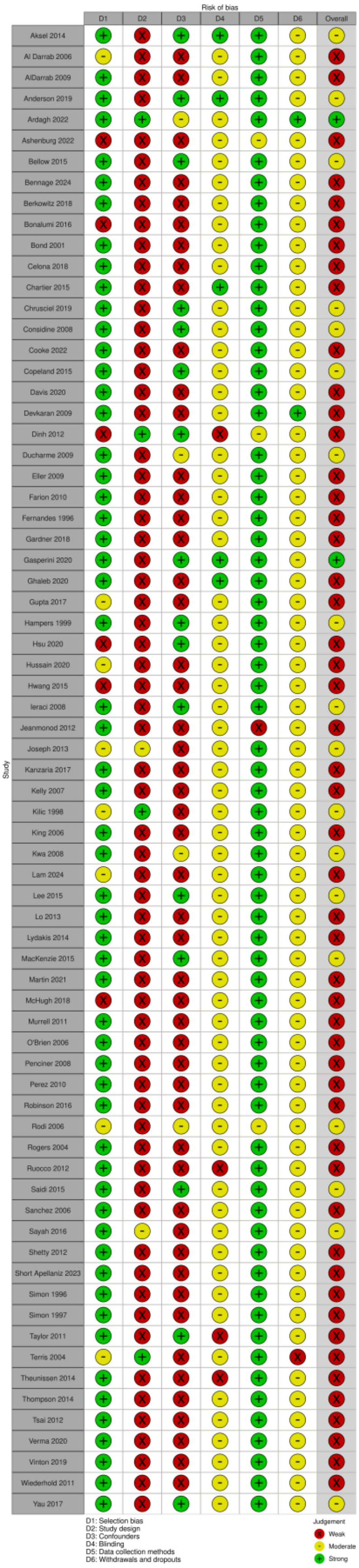

Figure 9: Risk of bias in UC streaming and combination studies

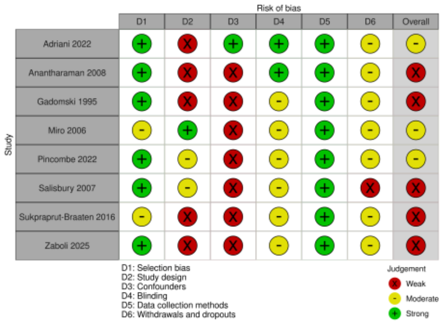

Figure 10: Risk of bias summary plot for all studies (all streaming types)

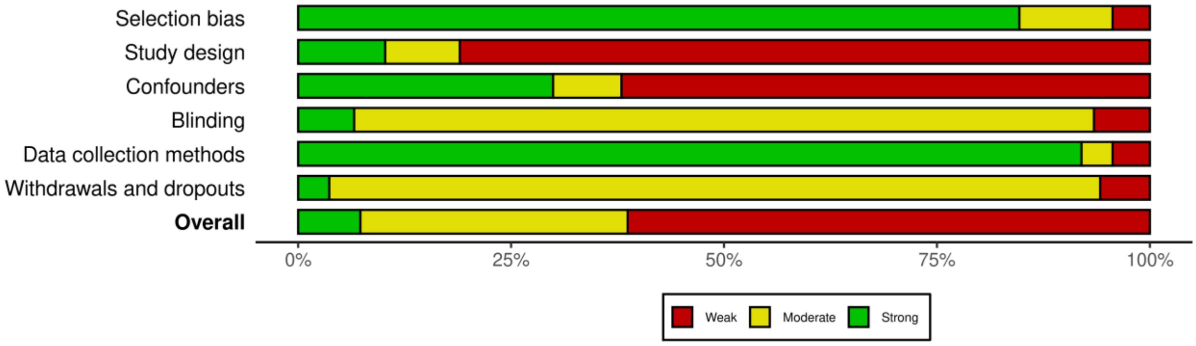

Supplement: Supplementary file 5 — Supplementary Material 5: Appendix 5 - Risk of bias.pdf. Plots showing Effective Public Health Practice Project tool ratings [file 12873_2026_1488_MOESM5_ESM.pdf]
