# Supplementary material for: Effectiveness and safety of emergency department-based streaming interventions for low-acuity utilizers - systematic review and meta-analysis
Source: BMC Emerg Med. 2026 Feb 19;26:58. doi: 10.1186/s12873-026-01488-w (PMC12922365; doi:10.1186/s12873-026-01488-w)

Appendix 6: Sensitivity analyses: separate analyses for studies with adult and pediatric populations

Forest plots in this Appendix illustrate independent analyses stratifying studies by population type. For explications of elements represented in the figures, see legends of the corresponding overall plots for each outcome in the main article.

Figure 11: Pooled proportions of alternative management – studies with adult populations

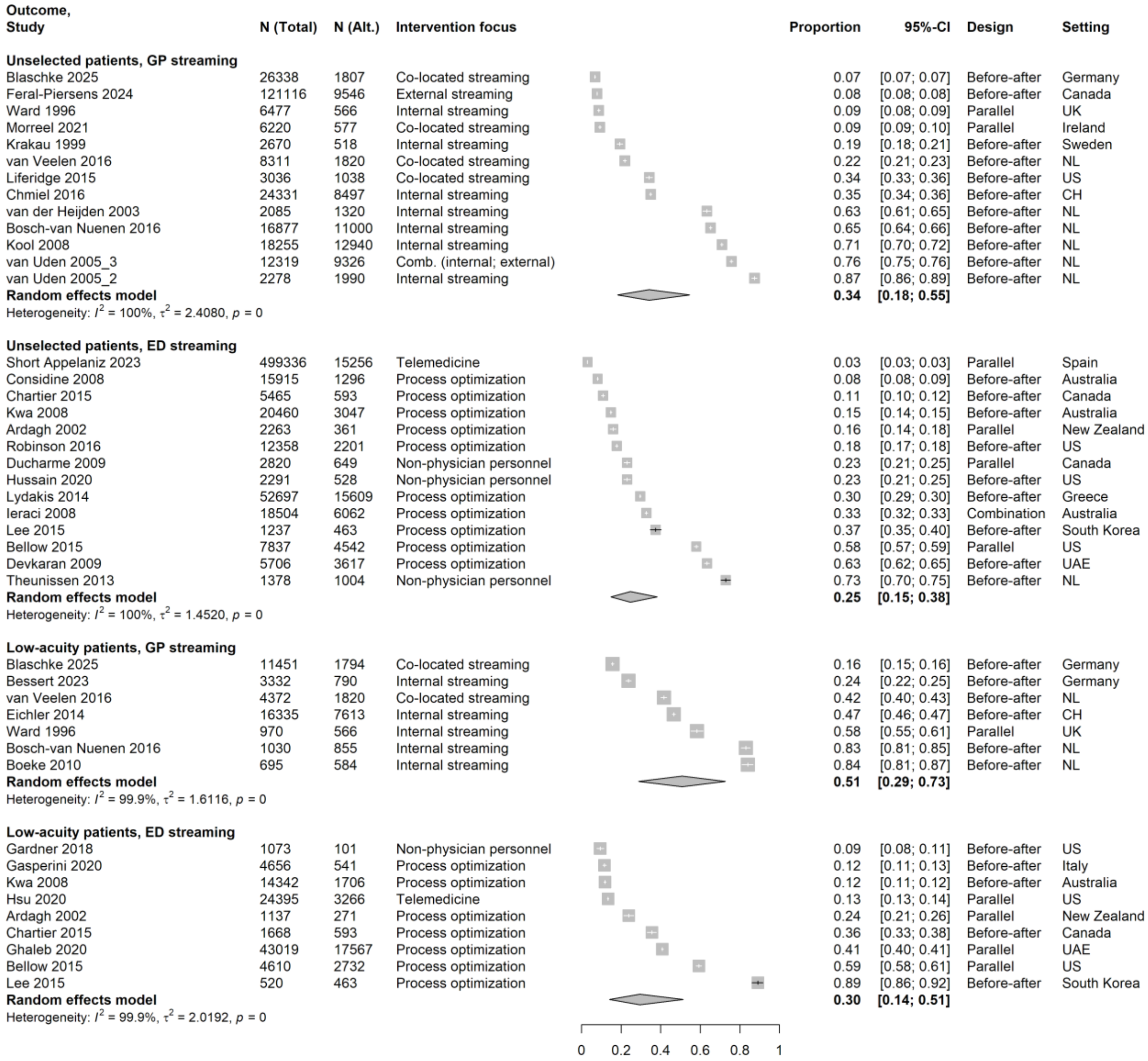

Figure 12: Pooled proportions of alternative management – studies with pediatric populations

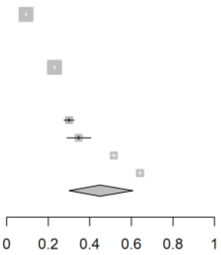

Outcome, Study	N (Total)	N (Alt.)	Intervention focus	Proportion	95%-CI	Design	Setting
**Unselected patients, GP streaming**							
Smith 2017	28655	2689	Internal streaming	0.09	[0.09; 0.10]	Parallel	UK
**Unselected patients, ED streaming**							
Tsai 2012	32053	7372	Process optimization	0.23	[0.23; 0.23]	Before-after	US
**Low-acuity patients, GP streaming**							
McCarron 2019	1393	418	Internal streaming	0.30	[0.28; 0.32]	Parallel	UK
van Veen 2012	275	95	External streaming	0.35	[0.29; 0.40]	Before-after	NL
Smith 2017	5223	2689	Internal streaming	0.51	[0.50; 0.53]	Parallel	UK
Leigh 2021	13099	8404	Internal streaming	0.64	[0.63; 0.65]	Parallel	UK
**Random effects model**				**0.45**	**[0.30; 0.61]**		
Heterogeneity: $I^2 = 99.6\%$, $\tau^2 = 0.4280$, $p < 0.0001$							

Figure 13: Effect sizes for waiting time – studies with adult populations

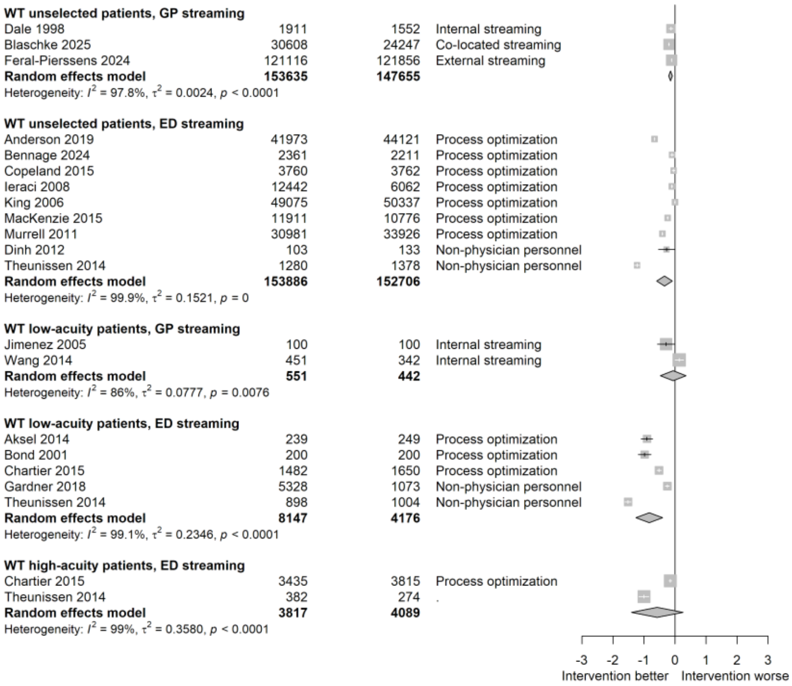

Outcome, Study	N (Control)	N (Intervention)	Intervention focus	SMD	95%-CI	Design	Setting
**WT unselected patients, GP streaming**							
Dale 1998	1911	1552	Internal streaming	-0.13	[-0.19; -0.06]	Parallel	UK
Blaschke 2025	30608	24247	Co-located streaming	-0.20	[-0.21; -0.18]	Before-after	Germany
Feral-Pierssens 2024	121116	121856	External streaming	-0.11	[-0.12; -0.10]	Before-after	Canada
**Random effects model**	**153635**	**147655**		**-0.15**	**[-0.20; -0.09]**		
Heterogeneity: $I^2 = 97.8\%$, $\tau^2 = 0.0024$, $p < 0.0001$							
**WT unselected patients, ED streaming**							
Anderson 2019	41973	44121	Process optimization	-0.66	[-0.67; -0.64]	Before-after	US
Bennage 2024	2361	2211	Process optimization	-0.09	[-0.15; -0.03]	Before-after	US
Copeland 2015	3760	3762	Process optimization	-0.05	[-0.09; -0.00]	Before-after	Canada
Ieraci 2008	12442	6062	Process optimization	-0.10	[-0.13; -0.07]	Parallel	Australia
King 2006	49075	50337	Process optimization	-0.00	[-0.01; 0.01]	Before-after	Australia
MacKenzie 2015	11911	10776	Process optimization	-0.24	[-0.26; -0.21]	Before-after	US
Murrell 2011	30981	33926	Process optimization	-0.41	[-0.42; -0.39]	Before-after	US
Dinh 2012	103	133	Non-physician personnel	-0.27	[-0.53; -0.01]	Parallel	Australia
Theunissen 2014	1280	1378	Non-physician personnel	-1.22	[-1.31; -1.14]	Before-after	NL
**Random effects model**	**153886**	**152706**		**-0.34**	**[-0.59; -0.08]**		
Heterogeneity: $I^2 = 99.9\%$, $\tau^2 = 0.1521$, $p = 0$							
**WT low-acuity patients, GP streaming**							
Jimenez 2005	100	100	Internal streaming	-0.29	[-0.56; -0.01]	Before-after	Spain
Wang 2014	451	342	Internal streaming	0.14	[-0.00; 0.28]	Before-after	CH
**Random effects model**	**551**	**442**		**-0.06**	**[-0.47; 0.36]**		
Heterogeneity: $I^2 = 86\%$, $\tau^2 = 0.0777$, $p = 0.0076$							
**WT low-acuity patients, ED streaming**							
Aksel 2014	239	249	Process optimization	-0.90	[-1.09; -0.72]	Before-after	Turkey
Bond 2001	200	200	Process optimization	-0.98	[-1.19; -0.77]	Before-after	Saudi Arabia
Chartier 2015	1482	1650	Process optimization	-0.51	[-0.59; -0.44]	Before-after	Canada
Gardner 2018	5328	1073	Non-physician personnel	-0.24	[-0.31; -0.18]	Before-after	US
Theunissen 2014	898	1004	Non-physician personnel	-1.51	[-1.61; -1.41]	Before-after	NL
**Random effects model**	**8147**	**4176**		**-0.83**	**[-1.26; -0.40]**		
Heterogeneity: $I^2 = 99.1\%$, $\tau^2 = 0.2346$, $p < 0.0001$							
**WT high-acuity patients, ED streaming**							
Chartier 2015	3435	3815	Process optimization	-0.15	[-0.20; -0.10]	Before-after	Canada
Theunissen 2014	382	274	.	-1.00	[-1.16; -0.84]	Before-after	NL
**Random effects model**	**3817**	**4089**	.	**-0.57**	**[-1.41; 0.26]**		
Heterogeneity: $I^2 = 99\%$, $\tau^2 = 0.3580$, $p < 0.0001$							

Figure 14: Effect sizes for waiting time – studies with pediatric populations

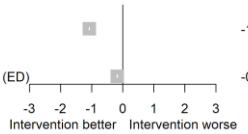

Outcome, Study	N (Control)	N (Intervention)	Intervention focus	SMD	95%-CI	Design	Setting
**WT unselected patients, ED streaming**							
Tsai 2012	28360	32053	Process optimization	-1.08	[-1.09; -1.06]	Before-after	US
**WT unselected patients, combination**							
Adriani 2022	14822	15585	Co-located (UC), process optim. (ED)	-0.18	[-0.20; -0.15]	Before-after	Italy

Figure 15: Effect sizes for length of stay – studies with adult populations

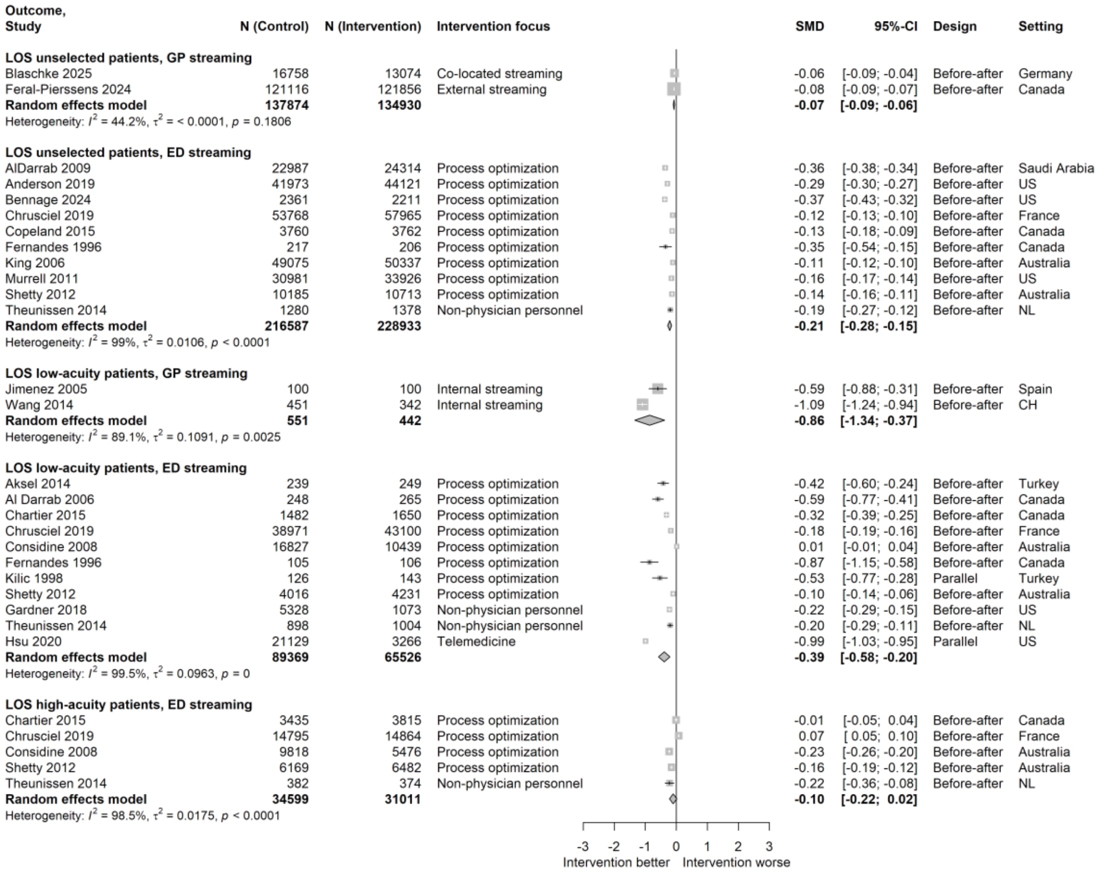

Figure 16: Effect sizes for length of stay – studies with pediatric populations

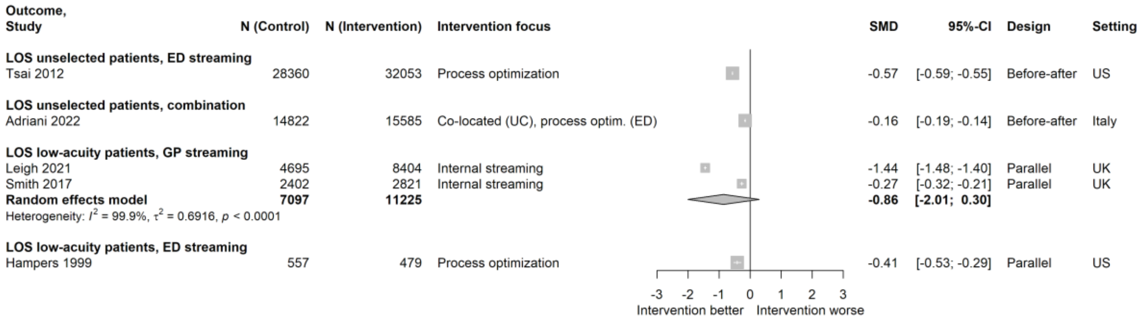

Figure 17: Effect sizes for hospital admissions – studies with adult populations

Outcome, Study	N (C)	N (I)	Rate (C)	Rate (I)	Intervention focus	OR	95%-CI	Design	Setting
**Hospital admissions, GP streaming**									
Bessert 2023	2377	2363	0.22	0.28	Internal streaming	1.41	[1.23; 1.61]	Before-after	Germany
Gibney 1999	1107	771	0.04	0.04	Internal streaming	1.12	[0.69; 1.80]	Parallel	Ireland
Harris 2014	134	250	0.38	0.40	Internal streaming	1.08	[0.71; 1.67]	Parallel	UK
Murphy 1996	2381	2303	0.10	0.04	Internal streaming	0.42	[0.33; 0.54]	Parallel	Ireland
Thijssen 2016	2285	1785	0.32	0.43	Internal streaming	1.61	[1.41; 1.83]	Parallel	NL
Wackers 2023	.	.			Internal streaming	1.07	[1.05; 1.10]	Parallel	NL
Agustin 1976	301473	351956	0.02	0.01	Co-located streaming	0.70	[0.67; 0.73]	Before-after	US
Morreel 2021	1784	6374	0.16	0.16	Co-located streaming	0.97	[0.84; 1.11]	Parallel	Belgium
Seeger 2017	160	164	0.07	0.00	Co-located streaming	0.04	[0.00; 0.62]	Before-after	Germany
Uthman 2018	10852	5426	0.20	0.06	Co-located streaming	0.24	[0.21; 0.27]	Parallel	UK
van Veelen 2016	7936	6322	0.17	0.25	Co-located streaming	1.60	[1.47; 1.73]	Before-after	NL
Feral-Pierssens 2024	121116	121856	0.16	0.15	External streaming	0.94	[0.92; 0.96]	Before-after	Canada
Davies 2024	1558841	2426062	0.37	0.23	Comb. (internal; co-located)	0.49	[0.49; 0.50]	Parallel	UK
Gaughan 2022	.	.			Comb. (internal; co-located)	1.03	[0.92; 1.16]	Parallel	UK
**Random effects model**	**2010447**	**2925633**				**0.83**	**[0.61; 1.14]**		
Heterogeneity: I^2 = 99.9%, τ^2 = 0.3247, p = 0									
**Hospital admissions, ED streaming**									
AlDarrab 2009	22987	24314	0.09	0.10	Process optimization	1.21	[1.14; 1.29]	Before-after	Saudi Arabia
Anderson 2019	43847	44792	0.17	0.17	Process optimization	1.01	[0.97; 1.04]	Before-after	US
Chrusciel 2019	53768	57965	0.28	0.26	Process optimization	0.91	[0.88; 0.93]	Before-after	France
Considine 2008	822	822	0.09	0.07	Process optimization	0.83	[0.59; 1.19]	Before-after	Australia
Copeland 2015	3575	3729	0.06	0.06	Process optimization	1.02	[0.84; 1.24]	Before-after	Canada
Ghaleb 2020	25452	11329	0.01	0.00	Process optimization	0.15	[0.09; 0.24]	Parallel	UAE
Ieraci 2008	12442	6062	0.43	0.11	Process optimization	0.17	[0.15; 0.18]	Parallel	Australia
Kelly 2007	31570	31515	0.24	0.23	Process optimization	0.97	[0.93; 1.01]	Before-after	Australia
Kwa 2008	18267	20460	0.23	0.21	Process optimization	0.92	[0.87; 0.96]	Before-after	Australia
Lee 2015	1116	1237	0.22	0.19	Process optimization	0.82	[0.67; 1.00]	Before-after	South Korea
Murrell 2011	30981	33926	0.13	0.12	Process optimization	0.89	[0.85; 0.93]	Before-after	US
Saidi 2015	.	.	0.17	0.16	Process optimization	0.93		Before-after	France
Dinh 2012	103	133	0.07	0.11	Non-physician personnel	1.61	[0.63; 4.16]	Parallel	Australia
Sanchez 2006	.	.	0.22	0.21	Non-physician personnel	0.98		Before-after	US
**Random effects model**	**244930**	**236284**				**0.73**	**[0.49; 1.09]**		
Heterogeneity: I^2 = 99.3%, τ^2 = 0.4913, p = 0									

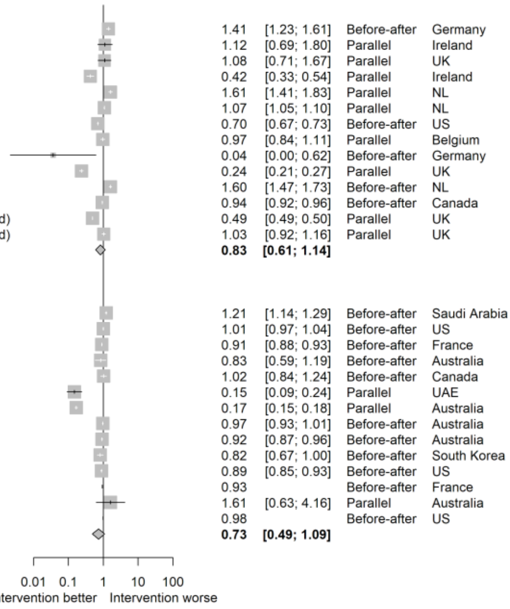

Figure 18: Effect sizes for hospital admissions – studies with pediatric populations

Outcome, Study	N (C)	N (I)	Rate (C)	Rate (I)	Intervention focus	OR	95%-CI	Design	Setting
**Hospital admissions, GP streaming**									
Leigh 2021	4695	8404	0.08	0.01	Internal streaming	0.16	[0.13; 0.20]	Parallel	UK
McCarron 2019	.	.	0.03	0.04	Internal streaming	1.12		Parallel	UK
Platter 2019	2133	1864	0.49	0.52	Internal streaming	1.12	[0.99; 1.27]	Before-after	NL
Smith 2017	2356	2821	0.06	0.03	Internal streaming	0.45	[0.34; 0.60]	Parallel	UK
van Veen 2012	1946	275	0.05	0.02	External streaming	0.41	[0.18; 0.94]	Before-after	NL
**Random effects model**	**11130**	**13364**				**0.43**	**[0.19; 0.98]**		
Heterogeneity: I^2 = 98.8%, τ^2 = 0.6531, p < 0.0001									
**Hospital admissions, ED streaming**									
Hampers 1999	557	479	0.02	0.00	Process optimization	0.09	[0.01; 0.67]	Parallel	US

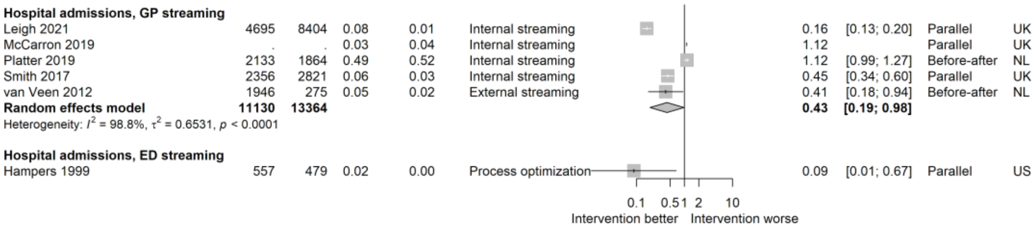

Figure 19: Effect sizes for patient safety outcomes (leaving without being seen and unplanned ED reattendances) – studies with adult populations

Outcome, Study	N (C)	N (I)	Rate (C)	Rate (I)	Intervention focus	OR	95%-CI	Design	Setting	Timeline
**LWBS, GP streaming**										
Bessert 2023	2377	2363	0.06	0.04	Internal streaming	0.55	[0.42; 0.72]	Before-after	Germany	.
Doran 2013	191	563	0.05	0.05	Co-located streaming	1.02	[0.47; 2.21]	Parallel	US	.
Uthman 2018	10852	5426	0.04	0.02	Co-located streaming	0.56	[0.45; 0.68]	Parallel	UK	.
Feral-Pierssens 2024	121116	121856	0.10	0.08	External streaming	0.80	[0.78; 0.82]	Before-after	Canada	.
Gaughan 2022	.	.			Comb. (internal; co-located)	0.91	[0.72; 1.16]	Parallel	UK	.
**Random effects model**	**134536**	**130208**				**0.71**	**[0.57; 0.90]**			
Heterogeneity: I^2 = 80.8%, τ^2 = 0.0486, p = 0.0003										
**LWBS, ED streaming**										
Aksel 2014	.	.	0.68	0.32	Process optimization	0.22		Before-after	Turkey	.
Al Darrab 2006	368	380	0.05	0.02	Process optimization	0.42	[0.19; 0.94]	Before-after	Canada	.
Anderson 2019	43847	44792	0.03	0.00	Process optimization	0.17	[0.15; 0.19]	Before-after	US	.
Bonalumi 2016	.	.	0.04	0.02	Process optimization	0.60		Before-after	US	.
Chrusciel 2019	53768	57965	0.10	0.05	Process optimization	0.51	[0.49; 0.54]	Before-after	France	.
Copeland 2015	3575	3729	0.05	0.04	Process optimization	0.96	[0.77; 1.20]	Before-after	Canada	.
Devkaran 2009	4479	5706	0.47	0.07	Process optimization	0.09	[0.08; 0.10]	Before-after	UAE	.
Gardner 2018	5328	1073	0.05	0.02	Process optimization	0.47	[0.30; 0.72]	Before-after	US	.
Gupta 2017	.	.	0.03	0.02	Process optimization	0.51		Before-after	US	.
Ieraci 2008	12442	6062	0.03	0.04	Process optimization	1.26	[1.06; 1.50]	Parallel	Australia	.
Kanzaria 2017	.	.	0.08	0.06	Process optimization	0.69		Before-after	US	.
Kelly 2007	31570	31515	0.04	0.04	Process optimization	1.00	[0.92; 1.09]	Before-after	Australia	.
King 2006	49075	50337	0.06	0.03	Process optimization	0.56	[0.53; 0.60]	Before-after	Australia	.
Lo 2013	12880	11640	0.02	0.01	Process optimization	0.45	[0.35; 0.58]	Before-after	US	.
MacKenzie 2015	11911	10776	0.03	0.01	Process optimization	0.24	[0.19; 0.31]	Before-after	US	.
Murrell 2011	30981	33926	0.04	0.01	Process optimization	0.32	[0.29; 0.36]	Before-after	US	.
Perez 2010	.	.	0.11	0.06	Process optimization	0.54		Before-after	US	.
Ruocco 2012	.	.	0.04	0.02	Process optimization	0.52		Before-after	US	.
Saidi 2015	.	.	0.14	0.09	Process optimization	0.61		Before-after	France	.
Shetty 2012	10185	10713	0.11	0.10	Process optimization	0.89	[0.81; 0.97]	Before-after	Australia	.
Taylor 2011	.	.	0.12	0.08	Process optimization	0.71		Before-after	Canada	.
Vinton 2019	.	.	0.02	0.02	Process optimization	1.00		Before-after	US	.
Wiederhold 2011	.	.	0.11	0.06	Process optimization	0.52		Before-after	US	.
Ducharme 2009	8931	1076	0.06	0.05	Non-physician personnel	0.74	[0.55; 0.99]	Parallel	Canada	.
Sanchez 2006	.	.	0.08	0.04	Non-physician personnel	0.46		Before-after	US	.
**Random effects model**	**279340**	**269690**				**0.47**	**[0.31; 0.70]**			
Heterogeneity: I^2 = 99.3%, τ^2 = 0.5636, p = 0										
**Unplanned reattendance, GP streaming**										
Murphy 2000	2263	2209	0.43	0.41	Internal streaming	0.92	[0.81; 1.03]	Parallel	Ireland	***
Blaschke 2024	5946	4949	0.04	0.05	Co-located streaming	1.09	[0.91; 1.31]	Before-after	Germany	*
Doran 2013	191	662	0.07	0.04	Co-located streaming	0.54	[0.27; 1.08]	Parallel	US	**
Uthman 2018	10852	5426	0.06	0.06	Co-located streaming	0.95	[0.83; 1.10]	Parallel	UK	*
Gaughan 2022	.	.			Comb. (internal; co-located)	0.97	[0.94; 1.00]	Parallel	UK	*
**Random effects model**	**19252**	**13246**				**0.97**	**[0.94; 1.00]**			
Heterogeneity: I^2 = 24.4%, τ^2 = < 0.0001, p = 0.2589										
**Unplanned reattendance, ED streaming**										
Chrusciel 2019	53768	57965	0.11	0.12	Process optimization	1.09	[1.06; 1.14]	Before-after	France	**
Gasperini 2020	504	504	0.06	0.01	Process optimization	0.14	[0.05; 0.36]	Before-after	Italy	**
Ghaleb 2020	25452	11329	0.04	0.02	Process optimization	0.50	[0.44; 0.58]	Parallel	UAE	*
Ieraci 2008	12442	6062	0.04	0.04	Process optimization	1.20	[1.03; 1.40]	Parallel	Australia	*
Taylor 2011	.	.	0.08	0.07	Process optimization	0.97		Before-after	Canada	*
Hussain 2020	1763	528	0.03	0.04	Non-physician personnel	1.26	[0.76; 2.10]	Before-after	US	.
Sanchez 2006	.	.	0.05	0.05	Non-physician personnel	0.99		Before-after	US	*
Ashenburg 2022	.	2232	0.12	0.10	Telemedicine	0.82		Parallel	US	*
Hsu 2020	21129	3266	0.03	0.03	Telemedicine	1.11	[0.91; 1.37]	Parallel	US	*
**Random effects model**	**115058**	**81886**				**0.77**	**[0.44; 1.36]**			
Heterogeneity: I^2 = 96.1%, τ^2 = 0.4605, p < 0.0001										

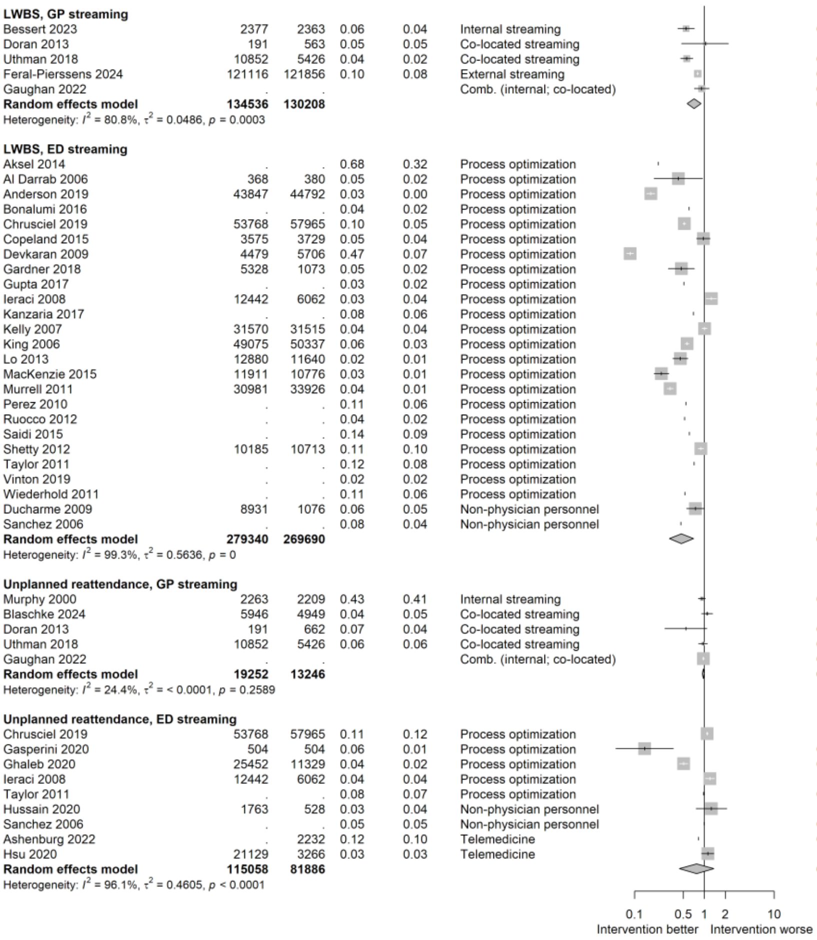

Figure 20: Effect sizes for patient safety outcomes (leaving without being seen and unplanned ED reattendances) – studies with pediatric populations

Outcome, Study	N (C)	N (I)	Rate (C)	Rate (I)	Intervention focus	OR	95%-CI	Design	Setting	Timeline
**LWBS, GP streaming**										
Leigh 2021	4695	8404	0.10	0.01	Internal streaming	0.11	[0.09; 0.14]	Parallel	UK	.
Smith 2017	2402	2821	0.06	0.03	Internal streaming	0.54	[0.41; 0.71]	Parallel	UK	.
**Random effects model**	**7097**	**11225**				**0.25**	**[0.05; 1.15]**			
Heterogeneity: I^2 = 98.7%, τ^2 = 1.2263, p < 0.0001										
**LWBS, ED streaming**										
Farion 2010	.	.	0.04	0.03	Process optimization	0.75		Before-after	Canada	.
Thompson 2014	.	.	0.01	0.00	Process optimization	0.38		Before-after	US	.
Tsai 2012	28360	32053	0.10	0.03	Process optimization	0.29	[0.27; 0.31]	Before-after	US	.
**LWBS, combination**										
Adriani 2022	14822	15585	0.13	0.06	Co-located (UC), process optim. (ED)	0.42	[0.39; 0.46]	Before-after	Italy	.
**Unplanned reattendance, GP streaming**										
McCarron 2019	.	.	0.10	0.07	Internal streaming	0.71		Parallel	UK	.
Platter 2019	888	716	0.04	0.04	Internal streaming	1.00	[0.62; 1.62]	Before-after	NL	.
**Unplanned reattendance, ED streaming**										
Hampers 1999	265	215	0.15	0.18	Process optimization	1.21	[0.75; 1.96]	Parallel	US	*

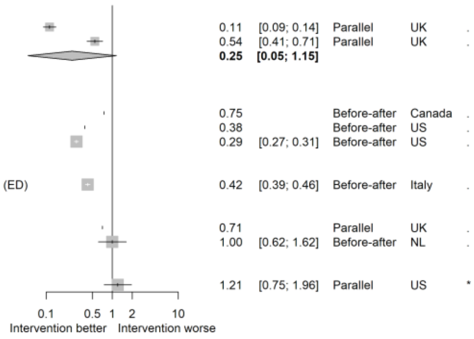

Supplement: Supplementary file 6 — Supplementary Material 6: Appendix 6 - Sensitivity analyses.pdf. Forest plots of separate analyses for studies with adult and pediatric populations [file 12873_2026_1488_MOESM6_ESM.pdf]
